# Pairing Mechanism for the High-T_C_ Superconductivity: Symmetries and Thermodynamic Properties

**DOI:** 10.1371/journal.pone.0031873

**Published:** 2012-04-18

**Authors:** Radosław Szczęśniak

**Affiliations:** Institute of Physics, Częstochowa University of Technology, Częstochowa, Poland; Lawrence Berkeley National Laboratory, United States of America

## Abstract

The pairing mechanism for the high-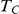 superconductors based on the electron-phonon (EPH) and electron-electron-phonon (EEPH) interactions has been presented. On the *fold* mean-field level, it has been proven, that the obtained *s*-wave model supplements the predictions based on the BCS van Hove scenario. In particular: (i) For strong EEPH coupling and 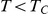 the energy gap (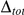) is very weak temperature dependent; up to the critical temperature 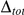 extends into the anomalous normal state to the Nernst temperature. (ii) The model explains well the experimental dependence of the ratio 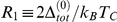 on doping for the reported superconductors in the terms of the few fundamental parameters. In the presented paper, the properties of the *d*-wave superconducting state in the two-dimensional system have been also studied. The obtained results, like for *s*-wave, have shown the energy gap amplitude crossover from the BCS to non-BCS behavior, as the value of the EEPH potential increases. However, for 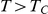 the energy gap amplitude extends into the anomalous normal state to the pseudogap temperature. Finally, it has been presented that the anisotropic model explains the dependence of the ratio 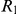 on doping for the considered superconductors.

## Introduction

In the study, we present the microscopic theory of the high-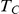 superconductivity [1], [2]. The organization of the paper is as follows:

In Section II (subsection 1) we call for the pairing mechanism. First of all, we discuss the main experimental and theoretical results. Next, on the basis of the presented analysis, we give the postulates, which determine the microscopic model for the high-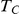 superconductors in the second quantization form.

In Section II (subsection 2), by using the unitary transformation, we deduce from the initial Hamiltonian the simple mean-field model. In the framework of the toy model (only the *s*-wave state), we discuss the properties of the energy gap in the superconducting and Nernst region; the numerical predictions are supplemented by the analytical approach. Next, for selected high-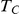 superconductors, we calculate the fundamental parameters of the model and compare the obtained theoretical results with the experimental data.

In Section II (subsection 3) we study the theromodynamic properties of the *d*-wave superconducting state in the two-dimensional system. We test the anisotropic model for the considered high-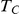 superconductors.

Finally, in Section III we summarize the results.

## Results

### The Pairing Mechanism

The real cuprates are three-dimensional. However, their physical properties are strongly anisotropic. On the basis of very small coherence length in the *c* direction (smaller than the interplane distance) one can suppose that the 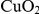 electrons play the special role in the physics of the high-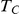 superconductors [3]. Unfortunately, the pairing mechanism for the planar problem remains highly controversial and many different hypotheses are suggested. In the literature two fundamental directions in search for the pairing mechanism have been crystallized. The first approach is based on the single-band Hubbard model, its extensions or related models *e.g.* 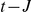 model [4], [5], [6], [7], [8], [9], [10], [11], [12]; the second approach emphasizes the relevance of the electron-phonon interaction [13], [14], [15].

Why is the Hubbard model so much studied? First of all, some analysis suggest that the one-band Hubbard model reproduces well the spectra of the more complicated three-band Hamiltonian for electrons in the copper oxide planes (the Emery model) [3], [5], [6]. For example, by using the finite cluster method, Hybertsen *et al.* have shown that the one-band Hubbard model with the small next-nearest-neighbor integral 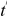 should have the following parameters: 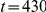 meV, 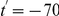 meV and 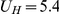 eV [16]. Secondly, for the half-filled electron band and large on-site Coulomb interaction, the Hubbard model reduces to the Heisenbeg model, which describes well the spin dynamics of the underdoped high-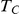 superconductors [3]. On the basis of the quoted facts some authors suppose, that the strong electronic correlations modeled by the Hubbard model can alone induce the superconducting state in the cuprates. Unfortunately, the studies carried out by several groups have shown that the Hubbard model gives no obvious evidence for superconductivity with the large critical temperature [17], [18], [19], [20]. On the other hand, there is the strong tendency for superconductivity in the attractive Hubbard model for the same value of the on-site Coulomb interaction. Finally, we noticed that probably also the three-band Hubbard model and the 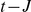 model do not superconduct at temperatures characteristic for the cuprates [3], [21].

The relevance of phonons to the pairing mechanism in the high-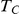 superconductivity also constitutes a complicated problem. On the one hand there exist many experimental observations which have been taken as evidence for the electron-phonon interaction in the cuprates. For example: the strong isotope effects on 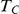 in the underdoped superconductors [22], the phonon renormalization in the Raman measurements [23], the phonon-related features of 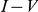 characteristics obtained by using the tunnelling experiments [24], [25], [26] and the dependence of the penetration depth on the substitution 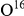 by 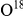 [27], [28]. Especially important results come from the ARPES measurements which give the evidence on the low-energy kink in the quasiparticle spectrum around the phonon energy both for the nodal and antinodal points [29], [30]; also the ARPES isotope effect in 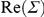 has been observed [31]. On the other hand the first principles calculations support the view that the conventional electron-phonon coupling is small [32]. For example, Bohnen *et al.* have predicted that the electron-phonon coupling constant for 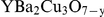 is equal to 0.27; so the strong Hubbard correlations should completely suppress the phonon-mediated superconductivity [33].

After summarizing the mentioned experimental and theoretical results, one can conclude that: (i) the cuprates belong to the strongly correlated systems but probably these correlations in the superconductivity domain are beyond the Hubbard or related approaches, since in these models the pairing correlations are too small, (ii) in the cuprates the conventional electron-phonon interaction is small but according to the experimental data one can suppose that the phonons play the important role in the pairing mechanism.

In order to solve the problem of high temperature superconductivity we present and examine the following scenario:

•(i) In the superconductivity domain of the cuprates the fundamental role is played by the electrons on the 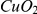 planes.
•(ii) In the cuprates exists the conventional electron-phonon interaction, which has not to be strong.
•(iii) In the cuprates exist strong electronic correlations, but the electron-electron scattering in the superconductivity domain is inseparably connected with the absorption or emission of vibrational quanta.

**Figure 1:**
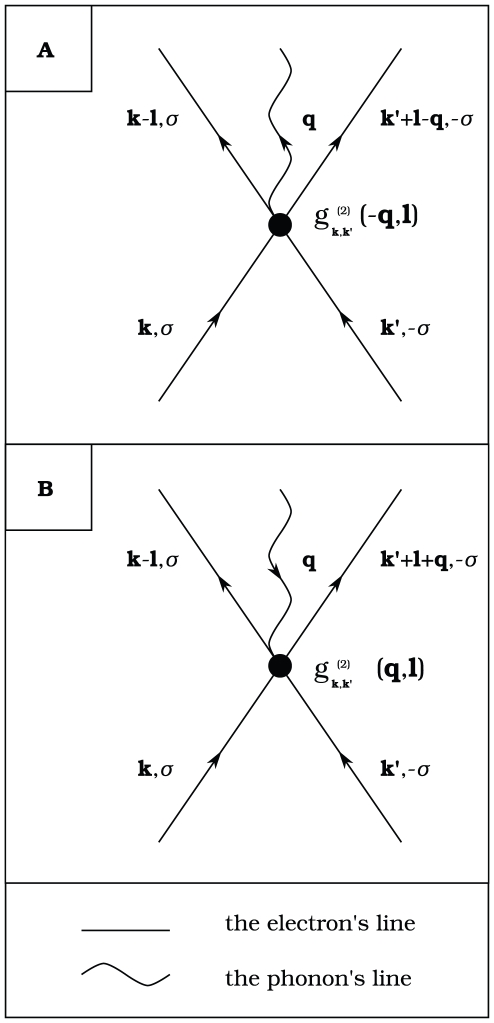
The graphical representation of the electron-electron-phonon coupling. The electron states are represented by the straight lines, while the phonon state by the curly line. The vertex is denoted by the black dot and its strength is given by 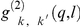. In Fig. 1 (A) the electrons emit the phonon during the scattering; in Fig. 1 (B) the electrons absorb the phonon.

In the simplest case the first and second postulate coincides with the phonon-van-Hove-scenario for high-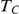 superconductors [34], [35]. The third postulate states that the effective electronic correlations in the superconductivity domain are connected with the three-body process: the electron-electron-phonon interaction. In Fig. 1 we show in detail the diagrammatic representation of this interaction. We notice that the EEPH coupling has a significant property which distinguish it from the Hubbard interaction; it does not destroy the classical phonon-mediated pairing correlations. Additionally, one should pay attention to the fact, that the first postulate has also the essential significance for the third postulate. Namely, for the two-dimensional case (the van Hove singularity at the Fermi level), the EEPH coupling has significantly strong influence on the physical properties of the system (see next subsection).

Below, we consider the Hamiltonian that describes the postulated coupling of the correlated electrons to phonons in the second quantization form:
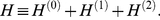
(1)


The first term represents the non-interacting electrons and phonons:
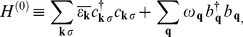
(2)where 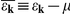; 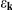 and 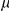 denotes the electron band energy and the chemical potential respectively. For the two-dimensional square lattice and the nearest-neighbor hopping integral *t*, we have: 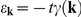, where 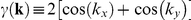. The symbol 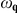 stands for the energy of phonons. The interaction terms are given by:
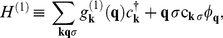
(3)and
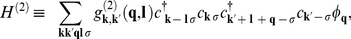
(4)where 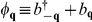. The matrix elements 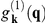 describe the electron-phonon interaction [36], [37] and the symbol 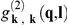 determines the strength of the electron-electron-phonon coupling. Since the Hamiltonian is the hermitian operator, we have: 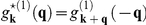 and 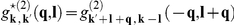.

### The Fold Mean-field Theory

From the mathematical point of view, the Hamiltonian (1) belongs to the class of so-called *dynamic Hubbard models*. These Hamiltonians describe modulation of the Hubbard on-site repulsion by the boson degree of freedom and were studied in detail by J.E. Hirch, F. Marsiglio and R. Teshima in the papers [38], [39], [40].

In the simplest case (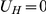), the dynamic Hubbard Hamiltonian describes the Holstein-like electron-boson interaction, where the coupling constant increases with the electron occupation. This model, however interesting, is inappropriate in the description of the high-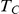 superconductivity. In the more elaborate scheme, the properties of the dynamic Hubbard model, can be studied by using the generalized Lang-Firsov transformation: 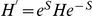, where the generator *S* has the form:


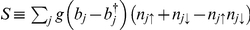
 and 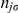 is the number operator for the site *j* and the spin 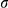. Next, the obtained effective Hamiltonian should be analyzed in the framework of the Eliashberg-like approximation [40], [41], [42], [43], [44], [45]; the central result is that the critical temperature increases with retardation.

We notice that the form of the Hamiltonan (1) suggests using the Eliashberg approach in order to analyse the physical properties of the considered system [42], [43], [44], [45]. This method is appropriate, because one can retain simultaneously the electron and phonon degrees of freedom. The detailed calculations in the framework of the Eliashberg approach the reader can to trace in: [46], [47], [48], [49], [50], [51], [52], [53], [54], [55]. Additionally, in the framework of the Eliashberg formalism, the formally exact expression for the self-energy matrix is easy to determine. The analysis of the above issue is presented in Appendix S1, where we have shown that: (i) in the case of the absence of the lattice distortion, the first-order contribution to the self-energy is equal to zero, (ii) the electron-phonon coupling is directly increased by the electron-electron-phonon interaction, (iii) the form of the second-order contributions to the matrix self-energy is very complicated. Unfortunately, this fact hampers considerably the detailed calculations on the Eliashberg level.

In the case of the presented paper, we have eliminated the phonon operators only from the EEFH term. The obtained Hamiltonian is next added to the BCS operator [56], [57]. As discussed in the following subsubsections, presented approximate description represents a generalization of the BCS van Hove scenario [35].

#### The unitary transformation

In order to eliminate the phonon degrees of freedom in the Hamiltonian (4) we use the Fröhlich-type unitary transformation:
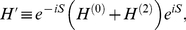
(5)where the operator *S* denotes the generator:
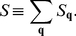
(6)In our case: 
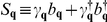
, where.
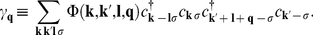
(7)


**Figure 2:**
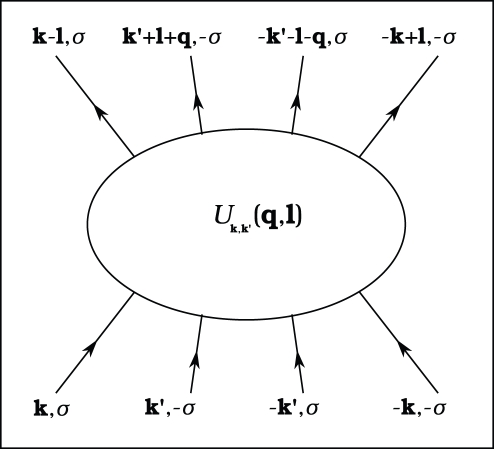
The four-body scattering event contributing to the interaction part of the Hamiltonian (10). The oval connecting the electron’s lines is an illustration of 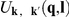 which denotes the effective potential.

The expression (5) can be rewritten in the approximate form:
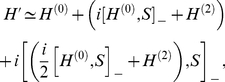
(8)where the square brackets 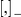 denote the commutator. To eliminate the second term in Eq. (8) we assume that the generator fulfills the relation: 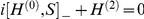, hence:



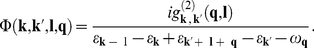
(9)Next, we can reduce the Hamiltonian 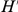 to the following expression:
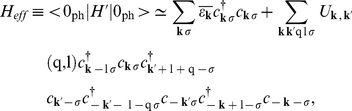
(10)where the phonon vacuum state is given by 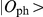. The pairing potential 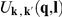 is of the form:



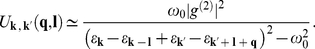
(11)We assume additionally that: 
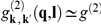
 and 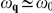; the symbol 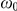 denotes the characteristic phonon frequency. On the basis of the expression (10) we conclude that the EEPH interaction can be replaced by the effective four electron-electron (4EE) scattering event; the diagrammatic representation of this interaction is shown in Fig. 2. From the Eq. (11) it is clear that the effective potential is attractive if: 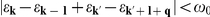. In the subsection, we consider the simplest case, when the attractive part of the 4EE potential can be written as:
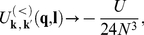
(12)for 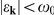. We have used the factor 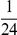, because the potential energy term represents the interaction between every four of particles counted once.

By using the *fold* mean-field (MF) approximation the Hamiltonian (10) takes the form (see also Appendix S2):
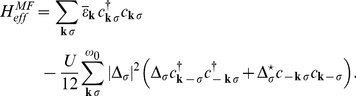
(13)


The symbol 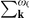 denotes the sum over the states when the 4EE potential is attractive;


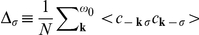


#### The toy model

The thermodynamic parameters of the high-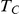 superconductors can have essentially different properties in comparison with the low-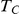 materials. From this reason, the analysis of the results obtained in the framework of the simplest approach is important, because these predictions facilitate the interpretation of the fundamental experimental data.

Taking into account the operator (13) we can write the total Hamiltonian in the form:
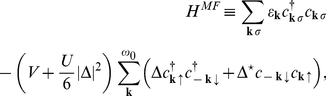
(14)where we have omitted the chemical potential and 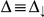. In Eq. (14), the symbol *V* represents the BCS pairing potential obtained from the Hamiltonian (3).

Now, we establish the energy scales in the presented model. The nearest-neighbor hopping integral *t* is of the order of (200–400) meV [58], [59], [60], [61]. We notice that in the numerical model calculations we take *t* as an energy unit. From the *ab initio* calculations arises the fact that 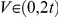 [33]. In order to determine qualitatively the possible values of *U*, we note, that the simple BCS pairing potential is obtained from the expression:
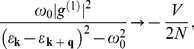
(15)where the characteristic phonon frequency 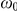 is of the order of Debye frequency (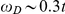); the electron-phonon coefficient 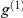 has nearly the same value [62]. Next, we assume that the largest energy in the high-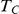 superconductors, of order (5–10) eV, is the electron-electron-phonon potential; in the other words 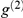 is comparable with the Coulomb repulsion in the one-band Hubbard model. Hence, 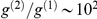. Then, by using Eqs. (11), (12) and (15) we can calculate the ratio 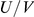. The result shows that U/V is proportional to 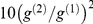. Due to this reason 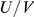 can be considerably larger than 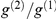.

From the mathematical point of view, the value of *U* is not as much important as the value of the mean-field potential (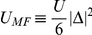). It is easy to see that, in contrast to the BCS pairing potential, 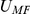 is 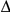-dependent (the fundamental feature of the presented model). So, the potential 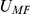 depends on the temperature, *V* and *U*. If we set *V* and *U* the mean-field potential reaches the maximum value for 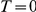 K: 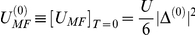, where the symbol 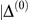 denotes the amplitude of the anomalous thermal average for 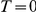 K. In the case of the analyzed superconductors, we have 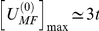 (see also next subsubsection).

By using the Hamiltonian (14) we calculate the anomalous Green function:
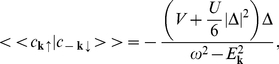
(16)where 
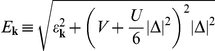
. The obtained propagator is more complicated than the BCS Green function; peculiarly, we draw the readers’ attention to the very intricate structure of the energy gap. Additionally, we notice that all omitted terms in anomalous Green function (in particular the terms linear with reference to 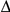 in the pairing potential) are unimportant since they are at most of 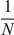 order (see Appendix S3). The fundamental equation can be found in the form:



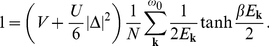
(17)In order to calculate the thermodynamic properties we transform the momentum summation over an energy integration in Eq. (17). We notice that, in the case of three-dimensional system, where the electron density of states near the Fermi energy is constant, the mean-field 4EE interaction can be neglected because the value of 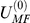 is very small. The situation changes dramatically for the two-dimensional system, where 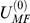 can be even greater than *V*. Then:
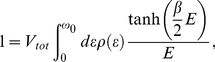
(18)where: 
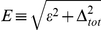
, 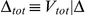 and 
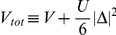
. In the case of the square lattice the density of states is given by [63], [64], [65], [66], [67], [68], [69], [70], [71], [72], [73]:
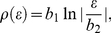
(19)where 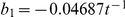 and 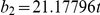.

**Figure 3:**
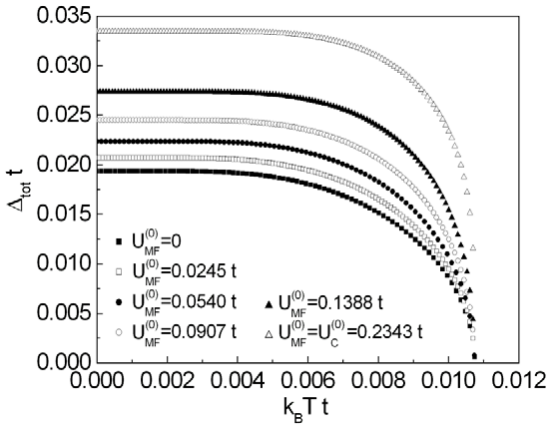
The dependence of 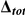 on the temperature for 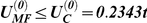. We assume 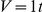 and 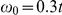.

**Figure 4:**
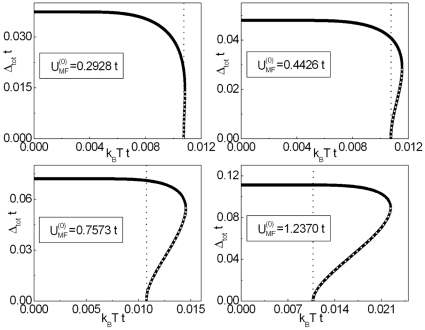
The dependence of 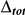 on the temperature for 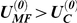. We assume 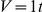 and 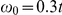. The vertical line indicates a position of the critical temperature. For 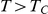 the solid line represents the higher branch, whereas the perforated line corresponds to the lower branch.

In Figs. 3 and 4 we show the numerical solutions of Eq. (18) for increasing values of 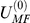. Analysis of the presented results allows one to state that only for 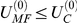 (where 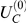 is some characteristic value) the gap equation has one solution. Above 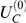 at 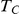 open the two new branches of the energy gap.

We notice that in the framework of the obtained mean-field model the 4EE interaction does not influence on the value of 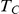; so, the critical temperature can be calculated by using the expression [63], [64]:
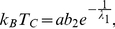
(20)where
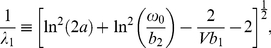
(21)and 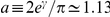 (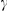 is the Euler constant). In this case the isotope coefficient is small and can be calculated from: 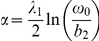.

**Figure 5:**
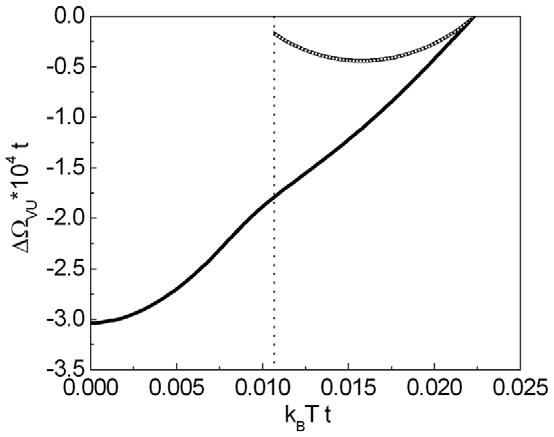
The dependence of 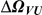 on the temperature for 
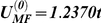
. We assume 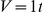 and 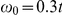. The vertical line indicates the position of the critical temperature. The solid line for 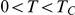 represents the result for the superconducting solution; the solid line for 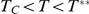 corresponds to the higher branch. The perforated line represents the thermodynamic potential for the lower branch. We notice that 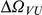 for the lower branch has the jump at 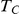. This behavior is connected with the inversion of the solutions of Eq. (18) in the considered region viz., for 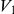 and 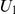 higher than 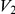 and 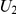 we have 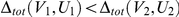.

Returning to the central line of the thought, it can be easily seen, that for both regions of 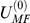 the values of 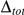 strongly increase when 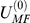 increases. For 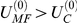 the evolution of the gap parameter with the temperature is sharply different from the classical BCS prediction (see Fig. 4 ). In particular for 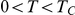, the superconducting gap is very weak temperature dependent; this anomalous behavior is frequently observed in the cuprates [74], [75] The another important results are presented for 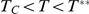, where 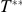 denotes the highest value of the temperature for which the non-zero solution of the gap equation exists. In this case we have two branches. In order to find out, for which of these solutions the thermodynamic potential is lower, the numerical calculations have been made. The detailed analysis of this complicated issue is presented in the Appendix S4 (see also Fig. S1). As an example, in Fig. 5 we show the dependence of the difference of the thermodynamic potential between the non-zero gap state and the normal state (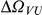) on the temperature. The obtained result proves, that the physical solution represents the higher branch; whereas the lower branch corresponds to the unstable state.

Next, the temperature 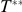 should be interpreted on the experimental background.

First, we notice that the complicated mathematical structure of the order parameter (in general, it is the complex function) imposes two conditions on the existence of the superconducting state: (i) the amplitude of the order parameter has to differ from zero and (ii) the superconducting state has to exhibit the long-range phase coherence.

The essential pointer, how should be interpreted the temperature 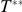, is connected with the experiments based on the Nernst effect [8], [76]. Namely, the Nernst signal above 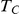 strongly suggests that the superconductivity vanishes at 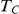 because the long-range phase coherence is destroyed by the thermally created vortices. Additionally, the experimental data have shown that the amplitude of the order parameter extends into the “normal” state to the temperature 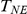 (the Nernst temperature). We notice that 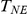 is considerably much lower than the pseudogap temperature (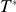). On the basis of presented experimental facts and the obtained theoretical results we assume that 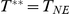.

**Figure 6:**
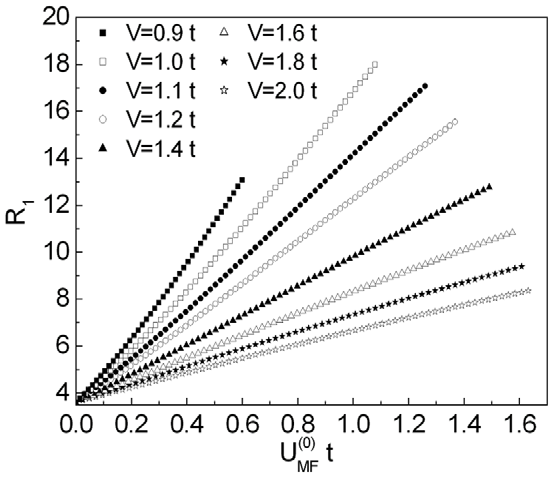
The dependence of the ratio 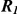 on 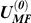 for different values of 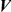. We assume 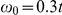. We notice that the ratio 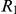 is plotted for different maximal values of 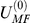, since this quantity is also strong dependent on *V*.

Before the comparison between the experimental data and the theoretical results obtained in the framework of toy model, we supplement the analytical approach. First, we notice that for 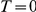 the integral equation (18) reduces to the algebraic equation:
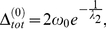
(22)where the symbol 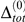 denotes the gap parameter at zero temperature and



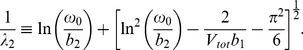
(23)By using the equation (22) and expression (20) one can easily calculate the ratio: 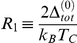. In the classical BCS theory 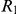 is the universal constant of the model and 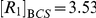. In the case of the BCS van Hove scenario 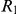 depends on the model parameters, however slightly (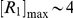). The results obtained in the framework of the toy model are shown in Fig. 6. We see that 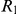 is always bigger than in the BCS van Hove scenario and for sufficiently big values of 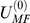 the ratio 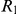 achieves the physically acceptable values.

Next, we consider the low-value behavior of the energy gap (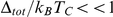). In this case, the equation (18) should be rewritten in the form:
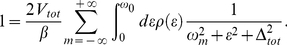
(24)


The kernel of the Eq. (24) may be expanded in powers of 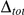:
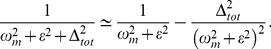
(25)


Next, we assume 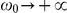, since 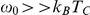. By using the lengthy but straightforward calculation we can transform the right-hand side of Eq. (24) into the algebraic form:
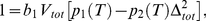
(26)where
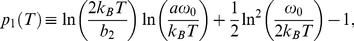
(27)and



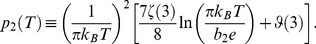
(28)The symbol 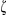 denotes the Riemann zeta function, 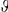 is defined by: 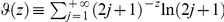. If we omit the terms higher as 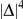 in Eq. (26), the expression for the anomalous thermal average takes the form:
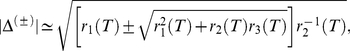
(29)where:



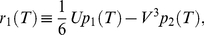
(30)

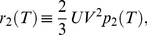
(31)and



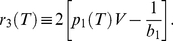
(32)We would like to point out that the expression (29) can be used only in the framework of the generalized mean-field model where 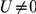; in the case of the BCS van Hove scenario the formula (29) generates the indeterminate form.

**Figure 7:**
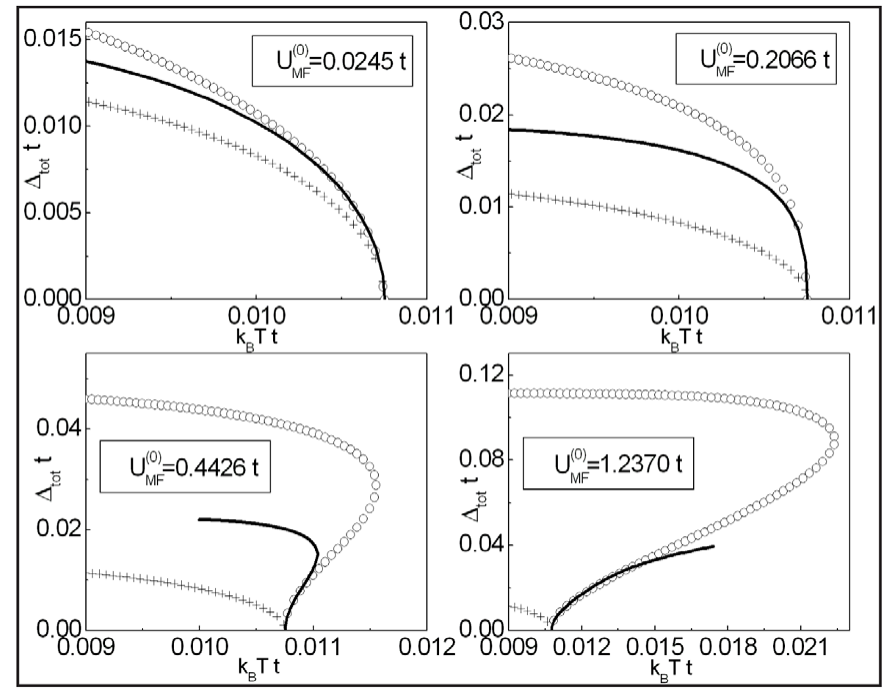
The dependence of 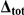 on the temperature close to the transition temperature for the small and large values of 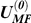. We assume 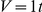 and 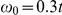. The empty circles correspond to the exact numerical calculations (Eq. (18) ). The solid line represents the calculation of 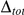 by using the formula (29). The crosses are obtained in the BCS van Hove scenario.

In Fig. 7 we illustrate the temperature dependence of the gap parameter close to 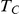 for different values of 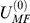. In particular, we have shown the results obtained by using Eqs. (18) and (29); for comparison we calculate also the gap parameter in the BCS van Hove scenario. One can see that the approximate formula (29) very well reconstructs the exact numerical results both for the small and large values of 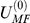; we strongly notice that for 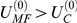 exists only the unstable state.

#### The toy model and the experimental results

The knowledge of the experimental values of 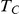, 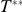 and other parameters (*t* and 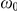) enables the calculation of *V* and 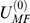 for the real materials. In particular, the value of *V* is obtained by using the expression (20). Next, the potential 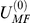 is determined from Eq. (18). We notice that, in principle, the quantities *V* and 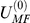 can be determined for the underdoped, optimally doped or overdoped samples; one can even take under consideration the influence of the disorder on *V* and 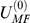. This is possible, since the strength of the presented approach lies in fact that the physical state of the high-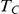 materials in the superconductivity domain is well reproduced by the values of 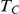 and 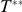. In general, the doping dependence can be calculated in the framework of the Eliashberg formalism. The initial study of this complicated issue has been presented in the paper [66].

In the subsubsection (for selected superconductors), on the basis of *V* and 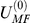, we calculate the dependence of the ratio 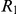 on the hole density or on the doping; we consider also the influence of the disorder on 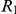. We compare the theoretical predictions with the experimental results (if the experimental data is known). In one case we plot openly the dependence of the energy gap on the temperature, since the right experimental data has existed in the literature.

In particular, we take under consideration following families of the high-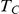 materials:


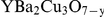
 (YBCO) - for selected values of the hole density (*p* - (holes/Cu)),


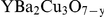
 (YBCO) - the disorder induced by electron irradiation, which results in the creation of point defects such as Cu and O vacancies in the 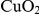 planes,


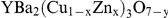
 (Zn-YBCO) - the in-plane disorder caused by zinc,


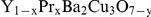
 (Pr-YBCO) - the out-of-plane disorder caused by praseodymium,


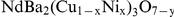
 (Ni-NdBCO) - for selected values of x and y.

Next, we consider 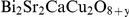 (Bi2212) - for selected values of the hole density, and 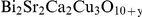 (Bi2223) - for the optimally doped case (OP).

Finally, 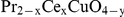 (PCCO) - the example of electron-doped cuprate.

The experimental data and the obtained theoretical results are collected in the Table 1. Mathematically, the range of the Nernst region can be characterized by the quantity: 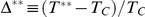. On the basis of experiments, we conclude that the Nernst region strongly expands if the hole density decreases; this is clear to see for YBCO and Bi2212, where 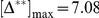 and 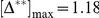 respectively; also for underdoped Ni-NdBCO, where 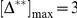. In the case of the disorder which is induced in YBCO, the value of 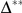 considerably increases if the electron irradiation is used or yttrium is substituted by praseodymium; the disorder caused by zinc weakly influences on the value of 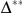. In the presented analysis we consider also the electron-doped cuprate system PCCO for which 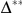 increases if doping decreases (from optimally to underdoped region). In overdoped region of PCCO the value of 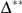 can not be determined, since 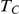 and 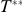 are experimentally indistinguishable.

**Table 1:** The quantities 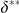, *V* and 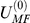 calculated by using the mean values of 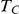 and 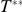.

Material	Type	*t*	Ref.	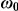	Ref.	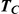	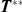	Ref.	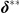	V	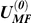
		meV		meV		K	K			t	t
YBCO	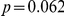	250	[59]	75	[33]	18.6	150.2	[121]	**7.08**	**0.838**	**2.847**
	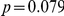					45	128.3		**1.85**	**1.148**	**2.045**
	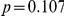					60.5	91.8		**0.52**	**1.297**	**1.171**
	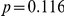					64.1	84.9		**0.32**	**1.330**	**0.969**
	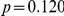					66.5	87.4		**0.31**	**1.352**	**0.974**
	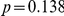					80.6	104.4		**0.30**	**1.475**	**1.062**
	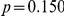					90	105		**0.17**	**1.554**	**0.898**
	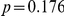					92	107	[76]	**0.16**	**1.571**	**0.903**
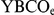	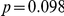	250	[59]	75	[33]	57	85	[122]	**0.49**	**1.265**	**1.104**
	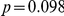					45.1	83.1		**0.84**	**1.150**	**1.288**
	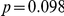					24.2	75		**2.10**	**0.915**	**1.564**
	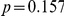					92.6	103		**0.11**	**1.576**	**0.796**
	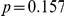					79.5	97.1		**0.22**	**1.465**	**0.930**
	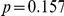					48.6	82.5		**0.70**	**1.184**	**1.213**
Zn-YBCO	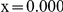	250	[59]	75	[33]	90	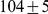	[123]	**0.16**	**1.554**	**0.876**
	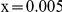					84	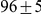		**0.14**	**1.504**	**0.813**
	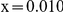					79	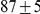		**0.10**	**1.461**	**0.700**
	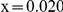					67	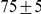		**0.12**	**1.356**	**0.674**
Pr-YBCO	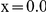	250	[59]	75	[33]	89.7	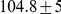	[124]	**0.17**	**1.552**	**0.899**
	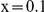					83.8	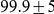		**0.19**	**1.502**	**0.908**
	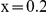					68.2	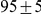		**0.39**	**1.367**	**1.096**
	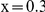					50.2	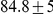		**0.69**	**1.200**	**1.226**
	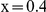					40.7	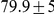		**0.96**	**1.104**	**1.313**
Ni-NdBCO	x = 0.00,y = 0.0	250	–	75	–	95	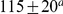	[125]	**0.21**	**1.596**	**1.021**
	x = 0.03,y = 0.0					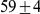	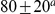		**0.36**	**1.283**	**0.966**
	x = 0.06,y = 0.0					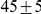	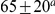		**0.44**	**1.148**	**0.940**
	x = 0.00,y = 0.2					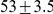	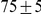		**0.42**	**1.227**	**0.981**
	x = 0.03,y = 0.2					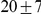	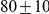		**3.00**	**0.858**	**1.737**
Bi2212	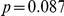	350	[126],[127],	80	[29],[30],	50	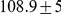	[76],[128].	**1.18**	**1.117**	**1.508**
	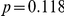		[129]		[31],[130],	77.9	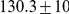		**0.67**	**1.347**	**1.442**
	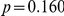				[131].	90.6	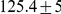		**0.38**	**1.444**	**1.184**
	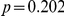					76.9	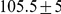		**0.37**	**1.339**	**1.050**
	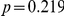					64.9	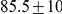		**0.32**	**1.243**	**0.888**
Bi2223	OP	350	–	80	–	109	135	[76]	**0.24**	**1.580**	**1.077**
PCCO	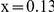	380	[60],[61],	33	[132],[133].	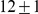	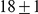	[134]	**0.50**	**0.795**	**0.715**
	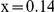		[135],[136].			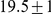	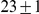		**0.18**	**0.947**	**0.529**
	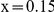					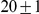	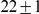		**–**	**0.956**	0.174
	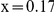					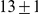	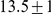		**–**	**0.817**	0.002
	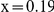					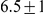	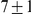		**–**	**0.654**	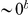

For YBCO the hole density *p* is estimated from the relation presented in [77]; for Bi2212 from the empirical formula 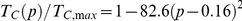 [120]. For the superconductors Ni-NdBCO and Bi2223 the values of *t* and 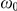 are unknown. In this case, we take *t* and 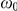 for YBCO and Bi2212 respectively.

a The high value of the experimental error.

b The value found based on 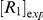.

Now, we consider the obtained values of *V* and 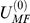. We notice that for real materials both *V* and 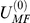 are significant and the following principle is in effect: if 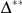 is smaller than 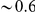 we have 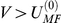, in the opposite case 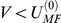; the especially high values of 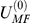 can be observed in the strongly underdoped regime.

By using the input parameters presented in Table 1 we compare the calculated theoretical values of the ratio 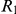 with the experimental data.

**Figure 8:**
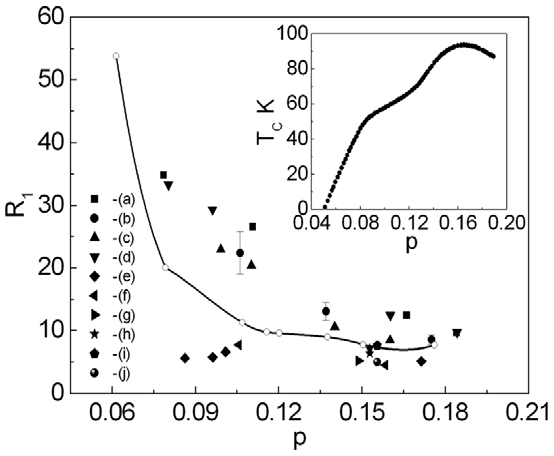
The dependence of the ratio 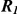 on *p* for YBCO. The solid line with the open circles represents the theoretical calculation. The black filled symbols correspond to experimental results obtained by: (a) - M. Sutherland *et al.* [84], (b) - K. Nakayama *et al.* [85], (c) - A. Kaminski *et al.* [86], (d) - M. Plate *et al.* [87], (e) - D.K. Morr *et al.* [88], H.F. Fong *et al.* [89], (f) - N.C. Yeh *et al.* [90], (g) - V. Born *et al.* [91], (h) - H. Murakami *et al.* [92], (i) - H. Edwards *et al.* [93], (j) - H. Edwards *et al.* [94]. The inset shows the dependence of the critical temperature on the hole density estimated from the relation presented in [77].

In Fig. 8 we show the ratio 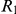 as a function of the hole density for YBCO. The numerical results are denoted by the solid line with the open circles; the experimental data by the black filled symbols (see also Appendix S5 and the Table S1). It can be seen that, from slightly underdoped to overdoped crystals the presented model correctly predicts the values of 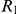 (taking under consideration the several approximations which have already been mentioned previously). For 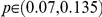 the theoretical values of the ratio 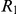 are probably lower than the experimental data. However, the increasing of 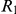 with decreasing of the hole concentration is qualitatively correctly predicted. In the case of the strongly underdoped crystals (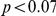) the toy model suggests very high values of the ratio 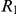 which have to be experimentally checked. In the inset in Fig. 8 there is presented the open dependence of the critical temperature on the hole density with help of which the values of *p* for YBCO have been calculated [77].

**Figure 9:**
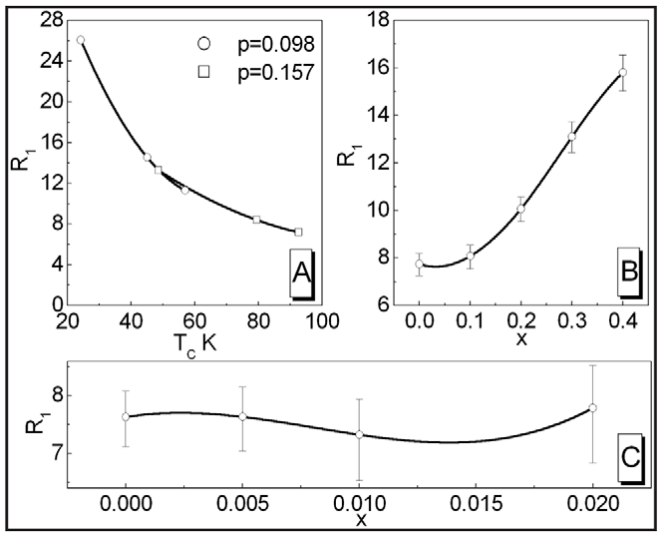
(*A*) The dependence of the ratio 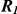 on 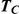 for 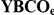; we consider the case 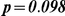 and 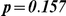. ( *B*) The dependence of the ratio 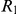 on x for Pr-YBCO. ( *C*) The dependence of the ratio 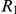 on x for Zn-YBCO.

In Figs. 9 (A)–(C) for YBCO we presented the influence of the disorder on the value of the ratio 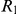. In Fig. 9 (A), the dependence of 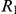 on 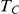 for the disorder induced by electron irradiation is shown. The two values of the hole concentration are considered: 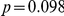 and 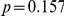. In both cases the growing disorder, which is being manifested by the lowering values of 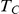, causes very distinct increase of the ratio 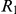. It is possible to observe the similar effect for the case, when the out-of-plane disorder is caused by praseodymium (Fig. 9 (B)). However, the in-plane disorder which is induced by zinc in principle doesn’t affect the value of the parameter 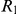; see Fig. 9 (C). In Figs. 9 (A)–(C) we have shown only the theoretical predictions, since the experimental data not yet exist.

**Figure 10:**
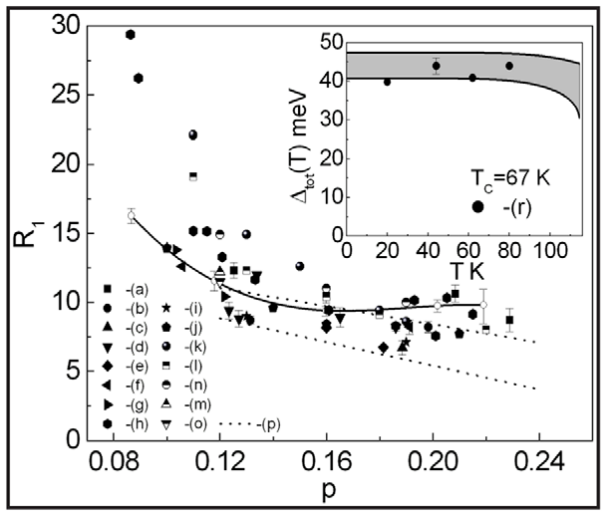
The dependence of the ratio 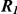 on *p* for Bi2212. The solid line with the open circles represents the theoretical calculation. The filled and half-filled symbols correspond to experimental results obtained by: (a) - Ch. Renner, *et al.* [74], [75], (b) - A. Hoffmann, *et al.* [95], (c) - Y.G. Ponomarev, *et al.* [96], (d) - T. Oki, *et al.* [97], (e) - V.M. Krasnov, *et al.* [98], (f) - A.K. Gupta, *et al.* [99], (g) - A. Kanigel, *et al.* [79], (h) - J.C. Campuzano, *et al.* [100], K. Tanaka, *et al.* [101], (i) - T. Nakano, *et al.* [102], (j) - M. Oda, *et al.* [103], (k) - K. McElroy, *et al.* [104], (l) - A. Matsuda, *et al.* [105], (m) - J.E. Hoffman, *et al.* [106], (n) - C. Howald, *et al.* [107], (o) - H. Murakami, *et al.* [108]. The lines (p) are obtained by using the empirical relation (33). The inset shows the dependence of the energy gap on the temperature; the filled region between the solid lines represents possible theoretical values of 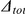; (r) - the experimental results [79].

The dependence of the ratio 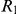 on the hole density for Bi2212 is presented in Fig. 10. Similarly as for YBCO, the theoretical results are denoted by the solid line with the open circles and the experimental data by the filled and half-filled symbols (Appendix S5 and the Table S2). Additionally, the region between the dotted lines represents the possible experimental values obtained by using the empirical formula [78]:
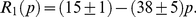
(33)


We can notice that relation (33) represents the linear least-squares fit to the experimental data and it is valid for 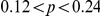. On the basis of the presented results we conclude, that the toy model, in a wide range of the *p* values, very correctly reproduces the experimental data. It is possible to have reservation only for very low values of *p*, where some experimental data suggested extremely high values of the ratio 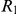. The inset in Fig. 10 presents the dependence of the energy gap on the temperature for Bi2212 with 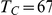 K. In particular, the region between the solid lines corresponds to the possible theoretical values of 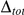. Let us notice that theoretical results were set with the experimentally accuracy of 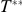 (see also the Table 1 ). In the inset we also show the experimental data obtained by A. Kanigel, *et al.* [79]. It is easy to see that the agreement between theoretical and experimental results is excellent.

However, for Bi2223 and Ni-NdBCO superconductors the values of *t* and 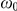 are unknown and we take *t* and 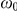 for Bi2212 and YBCO respectively, it is possible to receive good estimating of the real values of 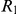. In the case of Bi2223 we have 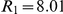. On the basis of the results presented in Appendix S5 and the Table S3 we see that our theoretical value is very close to the experimental data. For Ni-NdBCO the situation is more complicated. If we consider Ni-NdBCO superconductor with y = 0 the experimental error for 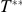 is too big, so it is impossible exactly determine 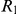. However, if we take the mean values of 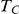 and 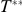 the theoretical results indicate that the ratio 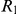 has the values 7.97, 10.20, 11.78 respectively for x = 0, x = 0.03 and x = 0.06. We notice that the experimental value of 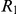 for x = 0 is equal to 7.3 (see Appendix S5 and the Table S4). For the case y = 0.2 the experimental data are much more accurate and we have 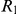 equal to 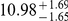 and 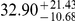 for x = 0 and x = 0.03 respectively. On the basis of above results we see that the last value of the ratio 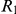 can be extraordinary big (this result should be checked experimentally).

The general phase diagram of the high-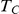 superconductors presents the global symmetry between the hole- and electron-doped materials [80]. First of all, in both cases, the antiferromagnetic phase has the similar Neel temperature (however for electron-doped cuprates, the antiferromagnetic phase is broader). Secondly, the superconducting phase for the hole- and electron-doped materials appears closely to antiferromagnetic phase, with the similar value of the optimal doping (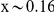). The distinct symmetry of the phase diagram strongly supports the view that the hole- and electron-doped superconductors should have an identical pairing mechanism. For that reason, the analysis of 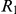 for the selected electron-doped superconductor in the framework of toy model is very important.

**Figure 11:**
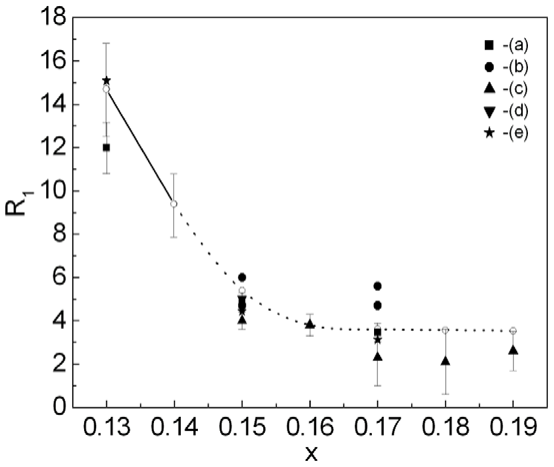
The dependence of the ratio 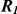 on x for PCCO. The solid line with the open circles represents the theoretical calculation based on 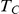 and 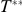; the dotted line with open circles represents the theoretical calculation based on 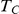 and the appropriately selected 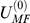. The black filled symbols correspond to experimental results obtained by: (a) - A. Biswas, *et al.* [109], (b) - A. Zimmers, *et al.* [110], (c) - Y. Dagan, *et al.* [111], (d) - C.C. Homes, *et al.* [112], (e) - P. Fournier, *et al.* [113].

In Fig. 11 we show the dependence of the ratio 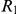 on doping for PCCO. The solid line with open circles represents the theoretical calculation obtained by using the input parameters 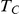 and 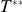; the dotted line with open circles represents the theoretical calculation obtained by using the input parameters 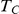 and the appropriately selected 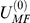. The black filled symbols correspond to experimental data (Appendix S5 and the Table S5). Based on Fig. 11, it is easy to see that the theoretical results very well reconstruct the experimental values of 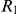.

### The Anisotropic Superconductivity

In the presented subsection we will study the thermodynamic properties of the *d*-wave superconducting state on the basis of the postulated pairing model. In particular, we will calculate the dependence of the energy gap amplitude 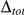 on the temperature for the selected values of the EEPH potential. Next, we will analyze the dependence of the 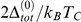 ratio on the hole density for 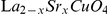 (LSCO) and Bi2212 superconductors. The theoretical predictions will be compared with the experimental data.

#### Model

The effective Hamiltonian we have rewritten in the form:
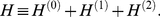
(34)


The first term represents the non-interacting electrons:
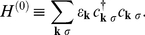
(35)


The EPH and EEPH interaction terms are given by:
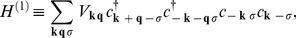
(36)and
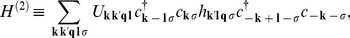
(37)where: 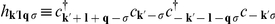. The functions 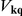 and 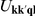 indicate the pairing potentials:
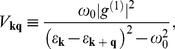
(38)and



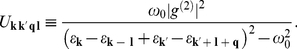
(39)On the basis of the operators (36) and (37) it is possible to deduce the Hamiltonians which describe the *d*-wave superconducting state. In the case of the Hamiltonian (36) this procedure is known and widely described in the literature (see e.g. the paper [81]). With reference to the above, we will discuss only the derivation of the anisotropic Hamiltonian on the basis of the EEPH operator. In the first step, we separate the momentums in the expression (37):
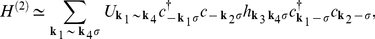
(40)where: 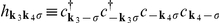.

With help of the relation: 
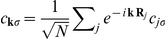
, we transform the operator (40) to the Wannier representation, where we restrict ourselves to on-site and the nearest neighbor pairing. The Hamiltonian takes the form:
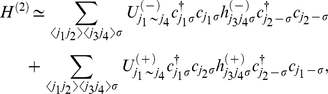
(41)where 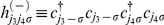 and 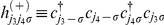. The symbols 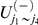 and 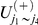 denote the local and kinetic potential respectively. Next, we return to the Bloch representation:
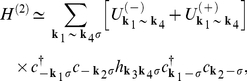
(42)where:



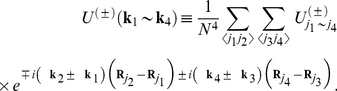
(43)Now, we assume: 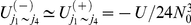, where the symbol 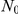 denotes the normalization factor: 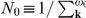. In the next step, we limit the symmetry of the energy gap to the dominating *d*-wave symmetry. The total Hamiltonian after applying the approximation presented for the *s*-wave takes the form:
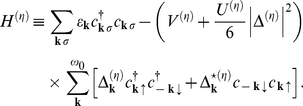
(44)


The symbol 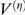 and 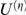 denotes the *d*-wave effective potential for the EPH and EEPH channel respectively. In particular: 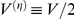 and 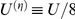. The anisotropic order parameter is given by: 
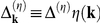
, where the amplitude is expressed as: 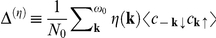 and 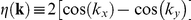.

On the basis of the Hamiltonian (44), we calculate the thermodynamic Green function by using the equation of motion method. The result has the form:
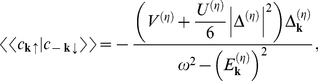
(45)where:



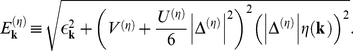
(46)We turn the reader’s attention toward the fact, that the obtained Green function possesses the analytical structure, which is more complex than the structure of the BCS Green function [56], [57] [82]. In particular, the energy gap amplitude is the complicated function of the order parameter amplitude. However, the energy gap, in spite of its complicated form, is characterized by the pure *d*-wave symmetry.

On the basis of Eq. (45) we derive the fundamental thermodynamic equation:
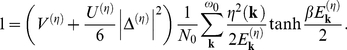
(47)


The sum 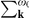 is approximated in the following manner: 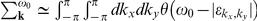, where the symbol 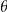 represents the unit step function. In the model calculations, we have taken *t* as the energy unit.

#### The numerical results

**Figure 12:**
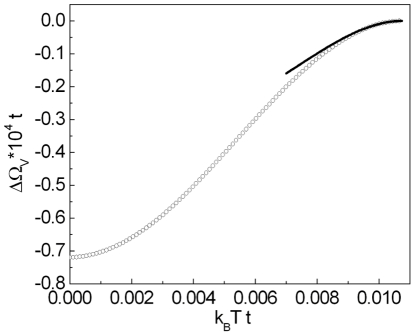
The dependence of the energy gap amplitude on the temperature for the selected values of the EEPH potential. The solid line represents the physical stable solution; the dotted line corresponds to the unstable solution, where the thermodynamic potential is bigger than in the first case. The vertical line indicates the position of the critical temperature.

In Fig. 12 we present the temperature dependence of the energy gap amplitude.

(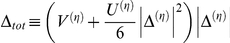) for 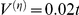, 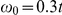 and the selected values of 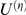. It is easy to see, that for the high values of the EEPH potential, the shape of the function 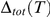 is sharply different from the BCS prediction. In particular, for 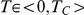 the energy gap is very weakly dependent on the temperature; up to the critical temperature 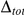 extends into the anomalous normal state to the temperature 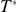. In the case of the *d*-wave superconducting state, the temperature 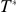 is interpreted as the pseudogap temperature (in contrast to the *s*-wave superconductivity, where the highest value of the temperature, for which the non-zero solution of the gap equation exists, is connected with the Nernst temperature 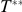).

Below we compare the theoretical predictions with the experimental data for LSCO and Bi2212 superconductors. For this purpose, we have calculated the values of the pairing potentials on the basis of 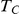 and 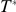 experimental values. The obtained results have been collected in Table 2. Next, by using the 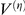 and 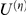 values, the hole density dependence of the 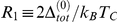 ratio has been obtained. We notice, that the energy gap amplitude at the temperature of zero Kelvin is defined as: 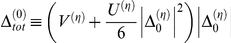, where 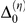 denotes the order parameter amplitude at the temperature of zero Kelvin.

**Table 2:** The parameters 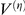 and 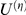 calculated by using 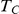 and the mean values of 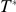.

Material	Type	*t*	Ref.	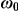	Ref.	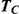	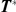	Ref.	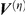	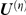
		meV		meV		K	K		meV	meV
LSCO	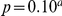	240	[58]	96	[13]	58	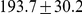	[114]	**3.76**	**66.86**
	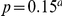					38	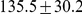	[114]	**4.41**	**53.89**
	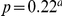					28	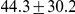	[114]	**3.91**	**45.11**
Bi2212	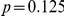	350	[126],[127],	80	[29],[30],	83	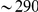	[74],[75]	**5.47**	**55.40**
	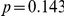		[129].		[31],[130],	90	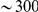	[137]	**5.68**	**56.37**
	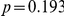				[131].	84	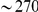	[137]	**5.51**	**51.72**
	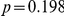					81	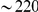	[137]	**5.40**	**43.88**

a The hole density *p* has been estimated as the doping 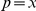.

**Figure 13:**
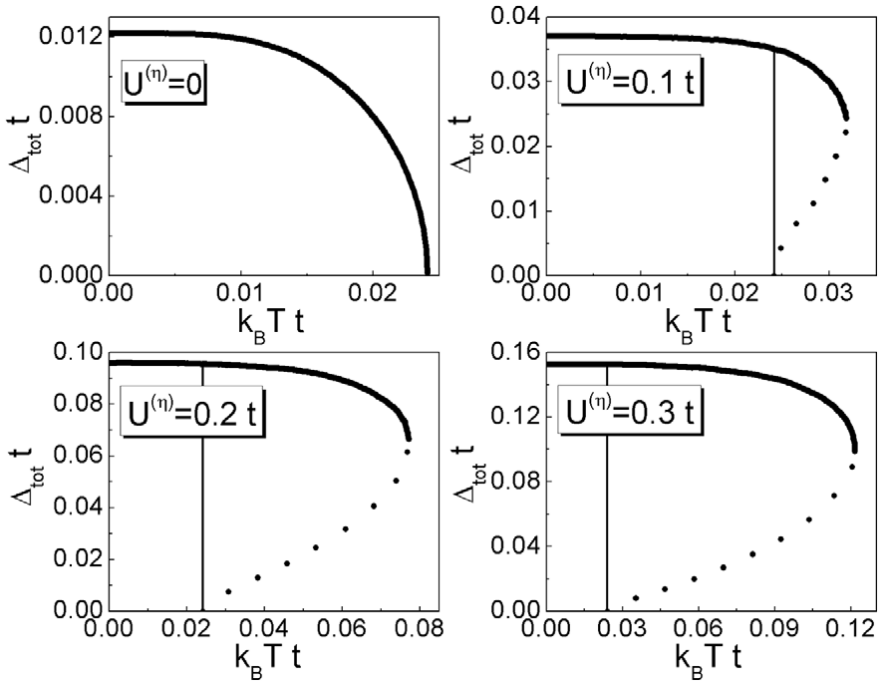
The ratio 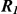 as a function of *p* for LCSO superconductor. The solid lines with the open squares represent the theoretical calculations based on the data presented in the paper [114]. The overshadowed areas mean the accuracy of the achieved results. The filled symbols correspond to the experimental results obtained by: (a) - Hashimoto, *et al.* [114], (b) - Nakano, *et al.* [102], (c) - Oda, *et al.* [115], (d) - Kato, *et al.* [116], (e) - Wang, *et al.* [117], (f) - Yoshida, *et al.* [118], (g) - Wen, *et al.* [119].

In Fig. 13 we present the dependence of the 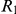 ratio on the hole density for LSCO superconductor. It can be seen, that with the increase of *p*, the parameter 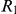 successively decreases. In particular, for the underdoped region (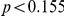) the values of 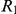 are significantly higher than the *d*-wave BCS value 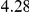 [83]. Slightly above 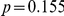 (the overdoped region) the 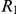 ratio approaches closely the weak-coupling *d*-wave BCS result. We notice, that for 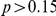, the lower accuracy of the theoretical results can not be determined, since 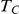 and 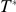 are experimentally indistinguishable (see Table 2 ). Now, we have compared the theoretical predictions which the experimental values of 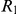, received by the few different researchers. The qualitative agreement of the theoretical predictions with the experimental data proves, that the measured dependence of the ratio 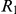 on *p* can be well reproduced with an use of the presented model.

**Figure 14:**
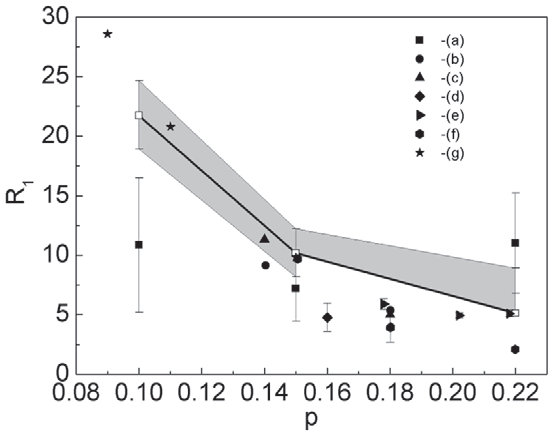
The dependence of the ratio 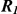 on *p* for Bi2212. The solid line with the open squares represents the theoretical calculation. The filled and half-filled symbols correspond to the experimental results obtained by: (a) - Renner, *et al.* [74], [75], (b) - Hoffmann, *et al.* [95], (c) - Ponomarev, *et al.* [96], (d) - Oki, *et al.* [97], (e) - Krasnov, *et al.* [98], (f) - Gupta, *et al.* [99], (g) - Kanigel, *et al.* [79], (h) - Campuzano, *et al.* [100], Tanaka, *et al.* [101], (i) - Nakano, *et al.* [102], (j) - Oda, *et al.* [103], (k) - McElroy, *et al.* [104], (l) - Matsuda, *et al.* [105], (m) - Hoffman, *et al.* [106]. The lines (n) are obtained by using the empirical relation (33).

In Fig. 14 we show the shape of the function 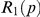 for Bi2212 superconductor. The presented results prove, that the theoretical line determines the high value of 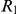 in the whole range of the considered hole density; 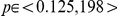. Important is also the fact, that the model correctly reconstructs the experimental data.

## Discussion

In the paper the microscopic model of the superconducting state that induces at high temperature was presented. The starting point of our considerations was the assumed statement: that a proper description of the superconducting condensate in the cuprates is possible only when the pairing mechanism would *inseparably* link together the strong electron correlations and the crystal lattice vibrations. The Hamiltonian proposed in the paper seems to be the most simple among the operators that satisfy the above postulate.

In the study we have shown, that for the large values of the EEPH coupling, the *s*-wave energy gap at the vicinity of the superconducting state existence weakly depends on the temperature and it vanishes above 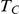 (at the temperature that was interpreted as Nernst temperature). The key test of our model was the determination of the dependence of the ratio 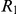 on doping for the selected superconductors. The obtained agreement between the theoretical and experimental data seems to be extraordinary, when taking into account the simplicity of the considered Hamiltonian and used approximations.

In the paper, we have also presented the model that describes the properties of the *d*-wave superconducting state in the two-dimensional system. In the first step, we have derived the fundamental thermodynamic equation. Next, on the basis of the exact numerical solution, we have shown, that for the high value of the EEPH potential, the temperature dependence of the energy gap amplitude differs sharply from the BCS prediction. In particular, the energy gap is slightly dependent on the temperature for 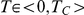; above the critical temperature, the energy gap persists to the pseudogap temperature. The theoretical predictions have been compared with the experimental data for LCSO and Bi2212 superconductors. It has been shown, that the calculated hole density dependence of the 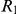 ratio correctly reproduces the experimental data.

In our opinion, the achieved results clearly suggest that presented pairing mechanism should be seriously taken into consideration in the further researches which may lead to the most precise understanding of the properties of the high temperature superconducting state.

## Methods

### Numerical Calculations

The calculations have been conducted on the Częstochowa University of Technology cluster, built in the framework of the PLATON project, no. POIG.02.03.00-00-028/08 - the service of the campus calculations U3. The Fortran programming language has been used.

## Supporting Information

Appendix S1 **The formally exact expression for the self-energy matrix.**
(PDF)

Appendix S2 **The** ***fold*** **mean-field approximation.**
(PDF)

Appendix S3 **The accuracy of the** ***fold*** **mean-field approximation in the framework of the random phase approximation (RPA) method.**
(PDF)

Appendix S4 **The van Hove and generalized mean-field thermodynamic potential.**
(PDF)

Appendix S5
**The lists of the experimental values of the thermodynamic parameters for the selected high-** 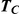 **superconductors.**
(PDF)

Figure S1
**The dependence of** 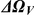 **on the temperature.** We assume 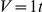 and 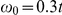. The empty circles are obtained from Eq. (8) with help of Eqs. (9) and (14). Solid line represents the calculation of 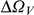 using the formula (16). We notice that the equations have been taken from the Appendix S4.(EPS)

Table S1 **The experimental data for YBa2Cu3O7−y (YBCO).**
(PDF)

Table S2 **The experimental data for Bi2Sr2CaCu2O8+y (Bi2212).**
(PDF)

Table S3 **The experimental data for Bi2Sr2Ca2Cu3O10+y (Bi2223).**
(PDF)

Table S4 **The experimental data for NdBa2Cu3O7−y (NdBCO).**
(PDF)

Table S5 **The experimental data for Pr2−xCexCuO4−y (PCCO).**
(PDF)
