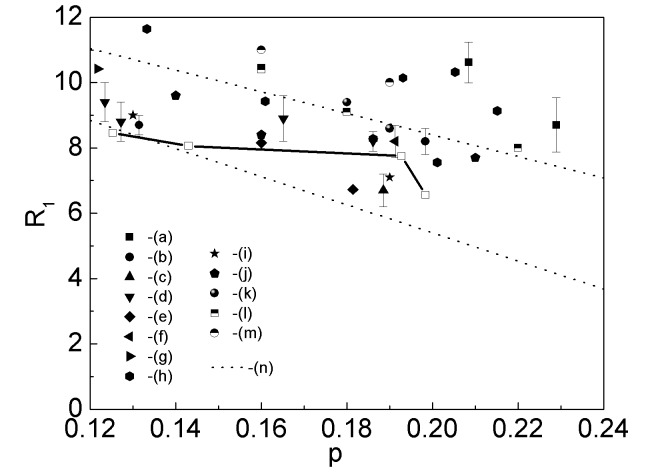# Correction: Pairing Mechanism for the High-T_C_ Superconductivity: Symmetries and Thermodynamic Properties

**DOI:** 10.1371/annotation/0ad3d34a-54c6-4b71-94df-80d32011fc2e

**Published:** 2012-06-01

**Authors:** Radosław Szczęśniak

There was an error in the placement of Figures 12, 13, and 14. The correct Figures can be veiwed here:

Figure 12: 

**Figure:**
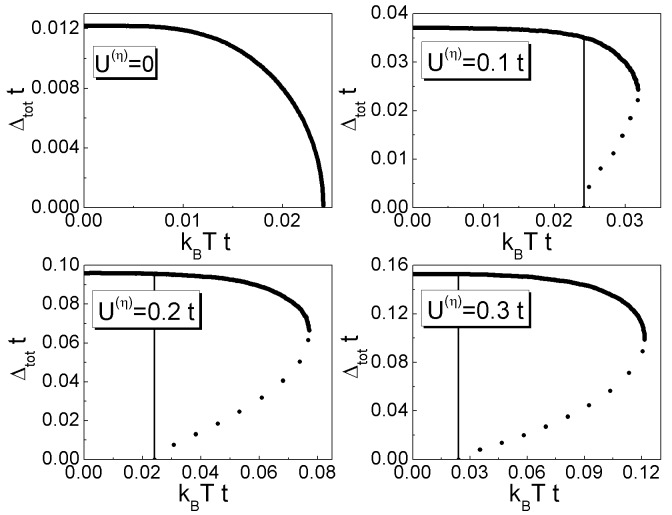


Figure 13: 

**Figure:**
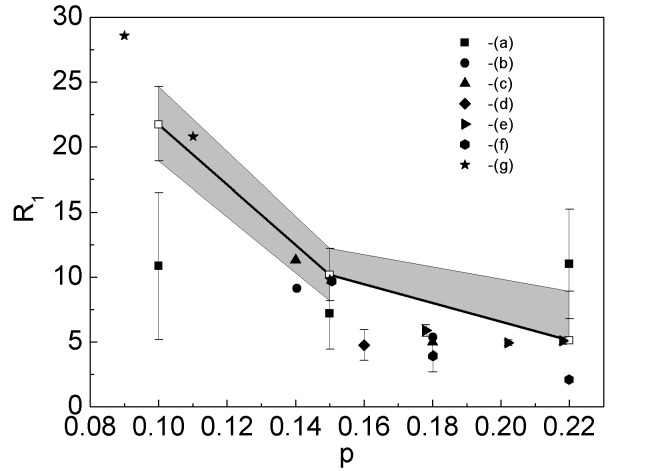


Figure 14: 

**Figure:**