# Supplementary material for: Pairing Mechanism for the High-TC Superconductivity: Symmetries and Thermodynamic Properties
Source: PLoS One. 2012 Apr 18;7(4):e31873. doi: 10.1371/journal.pone.0031873 (PMC3329537; doi:10.1371/journal.pone.0031873)
Supplement: Appendix S1 — The formally exact expression for the self-energy matrix. (PDF) [file pone.0031873.s001.pdf]

Appendix S1
Supporting information for

Pairing mechanism for the high-$\mathrm{T_C}$ superconductivity: symmetries and thermodynamic properties

Radosław Szczęśniak*
Institute of Physics, Częstochowa University of Technology, Al. Armii Krajowej 19, 42-200 Częstochowa, Poland
* E-mail: szczesni@wip.pcz.pl

The exact expression for the self-energy matrix

In the framework of the Eliashberg formalism, the Nambu spinors are defined in the following way:

$$\Psi_{\mathbf{k}} \equiv \left(\begin{array}{c} c_{\mathbf{k}\uparrow} \\ c^{\dagger}_{-\mathbf{k}\downarrow} \end{array} \right), \Psi^{\dagger}_{\mathbf{k}} \equiv \left(\begin{array}{cc} c^{\dagger}_{\mathbf{k}\uparrow} & c_{-\mathbf{k}\downarrow} \end{array} \right), \tag{1}$$

where $\Psi_{\mathbf{k}}$ and $\Psi^{\dagger}_{\mathbf{k}}$ satisfy the anticommutation rules:

$$\left[\Psi_{\mathbf{k}}, \Psi^{\dagger}_{\mathbf{k}'}\right]_{+} = \delta_{\mathbf{k}\mathbf{k}'}\tau_0. \tag{2}$$

In the presented notation, the terms of the Hamiltonian (1) in the main body of the paper can be rewritten as follows:

$$H^{(0)} = \sum_{\mathbf{k}} \overline{\varepsilon}_{\mathbf{k}} \Psi^{\dagger}_{\mathbf{k}} \tau_3 \Psi_{\mathbf{k}} + \sum_{\mathbf{q}} \omega_{\mathbf{q}} b^{\dagger}_{\mathbf{q}} b_{\mathbf{q}}, \tag{3}$$

$$H^{(1)} = \sum_{\mathbf{k}} g^{(1,2)}_{\mathbf{k}} \left(\mathbf{q}\right) \Psi^{\dagger}_{\mathbf{k}+\mathbf{q}} \tau_3 \Psi_{\mathbf{k}} \phi_{\mathbf{q}}, \tag{4}$$

and

$$\begin{aligned} H^{(2)} &= \sum_{\mathbf{k}\mathbf{k}'\mathbf{q}\mathbf{l}} g^{(2a)}_{\mathbf{k},\mathbf{k}'} \left(\mathbf{q},\mathbf{l}\right) \Psi^{\dagger}_{\mathbf{k}} \tau_B \Psi_{-\mathbf{k}'} \Psi^{\dagger}_{-\mathbf{k}'-\mathbf{l}+\mathbf{q}} \tau_C \Psi_{\mathbf{k}-\mathbf{l}} \phi_{\mathbf{q}} \\ &+ \sum_{\mathbf{k}\mathbf{k}'\mathbf{q}\mathbf{l}} g^{(2b)}_{\mathbf{k},\mathbf{k}'} \left(\mathbf{q},\mathbf{l}\right) \Psi^{\dagger}_{\mathbf{k}} \tau_C \Psi_{-\mathbf{k}'} \Psi^{\dagger}_{-\mathbf{k}'-\mathbf{l}+\mathbf{q}} \tau_B \Psi_{\mathbf{k}-\mathbf{l}} \phi_{\mathbf{q}}. \end{aligned} \tag{5}$$

The matrix elements take the form:

$$g^{(1,2)}_{\mathbf{k}} \left(\mathbf{q}\right) \equiv g^{(1)}_{\mathbf{k}} \left(\mathbf{q}\right) + g^{(2)}_{\mathbf{k}} \left(\mathbf{q}\right), \tag{6}$$

where:

$$g^{(2)}_{\mathbf{k}} \left(\mathbf{q}\right) \equiv \sum_{\mathbf{k}'} g^{(2)}_{\mathbf{k}',\mathbf{k}} \left(\mathbf{q},0\right) = \sum_{\mathbf{k}'} g^{(2)}_{\mathbf{k}',\mathbf{k}} \left(\mathbf{q},-\mathbf{q}\right), \tag{7}$$

and

$$g^{(2x)}_{\mathbf{k},\mathbf{k}'} \left(\mathbf{q},\mathbf{l}\right) \equiv \left\{ \begin{array}{ll} g^{(2)}_{\mathbf{k}-\mathbf{l},\mathbf{k}'+\mathbf{l}-\mathbf{q}} \left(\mathbf{q},-\mathbf{l}\right) & \text{for} \quad x=a \\ g^{(2)}_{-\mathbf{k},-\mathbf{k}'} \left(\mathbf{q},-\mathbf{l}\right) & \text{for} \quad x=b. \end{array} \right. \tag{8}$$

The bases of the matrices τ_A-τ_D and $\tau_0 - \tau_3$ (the Pauli matrices) are presented below:

$$\tau_A \equiv \begin{pmatrix} 1 & 0 \\ 0 & 0 \end{pmatrix} = \frac{1}{2}\left(\tau_0 + \tau_3\right), \tag{9}$$

$$\tau_B \equiv \begin{pmatrix} 0 & 1 \\ 0 & 0 \end{pmatrix} = \frac{1}{2}\left(\tau_1 + i\tau_2\right), \tag{10}$$

$$\tau_C \equiv \begin{pmatrix} 0 & 0 \\ 1 & 0 \end{pmatrix} = \frac{1}{2}\left(\tau_1 - i\tau_3\right), \tag{11}$$

$$\tau_D \equiv \begin{pmatrix} 0 & 0 \\ 0 & 1 \end{pmatrix} = \frac{1}{2}\left(\tau_0 - \tau_3\right). \tag{12}$$

With the help of definition (1) we have introduced the electron Green function $G_{\mathbf{k}}(i\omega_n) \equiv \left\langle\left\langle \Psi_{\mathbf{k}} | \Psi^{\dagger}_{\mathbf{k}} \right\rangle\right\rangle_{i\omega_n}$, where: $\omega_n \equiv (\pi/\beta)(2n-1)$ is the n-th Matsubara frequency and $\beta \equiv (k_B T)^{-1}$ (k_B is the Boltzmann constant). The electron propagator $G_{\mathbf{k}}(i\omega_n)$ can be written in the form:

$$G_{\mathbf{k}}(i\omega_n) = \begin{pmatrix} << c_{\mathbf{k}\uparrow} | c^{\dagger}_{\mathbf{k}\uparrow} >>_{i\omega_n} & << c_{\mathbf{k}\uparrow} | c_{-\mathbf{k}\downarrow} >>_{i\omega_n} \\ << c^{\dagger}_{-\mathbf{k}\downarrow} | c^{\dagger}_{\mathbf{k}\uparrow} >>_{i\omega_n} & << c^{\dagger}_{-\mathbf{k}\downarrow} | c_{-\mathbf{k}\downarrow} >>_{i\omega_n} \end{pmatrix}. \tag{13}$$

We notice that the superconducting thermal average should be evaluated from the non-diagonal part of $G_{\mathbf{k}}(i\omega_n)$, whereas the diagonal part determines the normal-state properties. It is essential, that the Hamiltonian in the matrix form enables to obtain the Dyson equation:

$$G_{\mathbf{k}}(i\omega_n) = G_{0\mathbf{k}}(i\omega_n) + G_{0\mathbf{k}}(i\omega_n) M_{\mathbf{k}}(i\omega_n) G_{0\mathbf{k}}(i\omega_n), \tag{14}$$

where $G_{0\mathbf{k}}(i\omega_n)$ denotes the unperturbed ($g^{(1)}_{\mathbf{k}}(\mathbf{q}) = g^{(2)}_{\mathbf{k},\mathbf{k}'}(\mathbf{q},\mathbf{l}) = 0$) propagator:

$$G_{0\mathbf{k}}(i\omega_n) \equiv \left(i\omega_n \tau_0 - \bar{\varepsilon}\tau_3\right)^{-1}, \tag{15}$$

and the self-energy matrix has the form:

$$\begin{aligned} M_{\mathbf{k}}(i\omega_n) \equiv\; & \left\langle \left[\left[\Psi_{\mathbf{k}}, H^{(2)} \right]_{-}, \Psi^{\dagger}_{\mathbf{k}} \right]_{+} \right\rangle \\ +\; & << \sum_{\mathbf{q}} g^{(1,2)}_{\mathbf{k}-\mathbf{q}}(\mathbf{q})\, \tau_3 \Psi_{\mathbf{k}-\mathbf{q}} \phi_{\mathbf{q}} | \sum_{\mathbf{q}'} g^{(1,2)}_{\mathbf{k}}\left(\mathbf{q}'\right) \Psi^{\dagger}_{\mathbf{k}+\mathbf{q}'} \phi_{\mathbf{q}'} \tau_3 >>_{i\omega_n} \\ +\; & << \sum_{\mathbf{q}} g^{(1,2)}_{\mathbf{k}-\mathbf{q}}(\mathbf{q})\, \tau_3 \Psi_{\mathbf{k}-\mathbf{q}} \phi_{\mathbf{q}} | \left[H^{(2)}, \Psi^{\dagger}_{\mathbf{k}} \right]_{-} >>_{i\omega_n} \\ +\; & << \left[\Psi_{\mathbf{k}}, H^{(2)} \right]_{-} | \sum_{\mathbf{q}'} g^{(1,2)}_{\mathbf{k}}\left(\mathbf{q}'\right) \Psi^{\dagger}_{\mathbf{k}+\mathbf{q}'} \phi_{\mathbf{q}'} \tau_3 >>_{i\omega_n} \\ +\; & << \left[\Psi_{\mathbf{k}}, H^{(2)} \right]_{-} | \left[H^{(2)}, \Psi^{\dagger}_{\mathbf{k}} \right]_{-} >>_{i\omega_n}. \end{aligned} \tag{16}$$

The equation (16) represents the formally exact expression for the self-energy matrix. The first-order contribution to $M_{\mathbf{k}}(i\omega_n)$ comes from the thermal average $\langle\rangle$ and the second-order contribution from the propagators in angular brackets. The complete analysis of this type of the matrix self-energy is very difficult problem [1]. However, one can demonstrate that, in the case of the lattice distortion's absence ($\langle b_{\mathbf{q}} \rangle = 0$), the first-order contribution to the self-energy is equal to zero. In particular:

$$\left\langle \left[\left[\Psi_{\mathbf{k}}, H^{(2)} \right]_{-}, \Psi^{\dagger}_{\mathbf{k}} \right]_{+} \right\rangle = \left\langle \left[\left[\Psi_{\mathbf{k}}, \widetilde{H}^{(2)} \right]_{-}, \Psi^{\dagger}_{\mathbf{k}} \right]_{+} \right\rangle \left\langle b^{\dagger}_{-\mathbf{q}} + b_{\mathbf{q}} \right\rangle = 0, \tag{17}$$

where $\widetilde{H}^{(2)}$ represents the fermionic parts in the Hamiltonian $H^{(2)}$. We notice that the non-diagonal part of the expression (17) is proportional to the superconducting thermal average, whereas the diagonal part is connected with the average number of electrons per lattice site.

The standard electron-phonon self-energy is represented by the second term in Eq. (16), which is proportional to $\left(g^{(1,2)}_{\mathbf{k}}(\mathbf{q})\right)^2$. On the basis of Eqs. (6) and (7) it is easy to see that the value of the electron-phonon coupling can be directly increased by the electron-electron-phonon interaction. One should clearly emphasize the fact that the existence of the $g^{(2)}_{\mathbf{k}}(\mathbf{q})$ element is the direct consequence of the form of the Hamiltonian in the symmetric Nambu notation. From the physical point of view, the above result is highly nontrivial. On the other hand, the remaining terms also give the important contributions to the electron-phonon pairing and, on the Eliashberg equations level, they can not be neglected.

References

1. Szczęśniak R (2009) Sdw antiferromagnetic phase in the two-dimensional hubbard model: Eliashberg approach. Phys Lett A 373: 473-479.
