# Supplementary material for: Pairing Mechanism for the High-TC Superconductivity: Symmetries and Thermodynamic Properties
Source: PLoS One. 2012 Apr 18;7(4):e31873. doi: 10.1371/journal.pone.0031873 (PMC3329537; doi:10.1371/journal.pone.0031873)
Supplement: Appendix S2 — The fold mean-field approximation. (PDF) [file pone.0031873.s002.pdf]

Appendix S2
Supporting information for

Pairing mechanism for the high-$\mathrm{T_C}$ superconductivity: symmetries and thermodynamic properties

Radosław Szczęśniak∗
Institute of Physics, Częstochowa University of Technology, Al. Armii Krajowej 19, 42-200 Częstochowa, Poland
∗ E-mail: szczesni@wip.pcz.pl

The *fold* mean-field approximation of the 4EE Hamiltonian

We have rewritten the interaction term in the Hamiltonian (10) in the main body of the paper:

$$H_{int} \equiv -\frac{U}{24N^3}\sum_{\mathbf{k}\mathbf{k}'\mathbf{q}\mathbf{l}\sigma}^{\omega_0} c^{\dagger}_{\mathbf{k}-\mathbf{l}\sigma}c_{\mathbf{k}\sigma}h^{(1)}_{\mathbf{k}'\mathbf{l}\mathbf{q}\sigma}c^{\dagger}_{-\mathbf{k}+\mathbf{l}-\sigma}c_{-\mathbf{k}-\sigma}, \tag{1}$$

where: $h^{(1)}_{\mathbf{k}'\mathbf{l}\mathbf{q}\sigma} \equiv c^{\dagger}_{\mathbf{k}'+\mathbf{l}+\mathbf{q}-\sigma}c^{\dagger}_{-\mathbf{k}'-\mathbf{l}-\mathbf{q}\sigma}c_{-\mathbf{k}'\sigma}c_{\mathbf{k}'-\sigma}$. By using the well known expression: $AB \simeq \langle A\rangle B + A\langle B\rangle - \langle A\rangle\langle B\rangle$, we have obtained:

$$h^{(1)}_{\mathbf{k}'\mathbf{l}\mathbf{q}\sigma} \simeq \Delta_{\mathbf{k}'\sigma}c^{\dagger}_{\mathbf{k}'+\mathbf{l}+\mathbf{q}-\sigma}c^{\dagger}_{-\mathbf{k}'-\mathbf{l}-\mathbf{q}\sigma} + \Delta^{\star}_{\mathbf{k}'+\mathbf{l}+\mathbf{q}\sigma}c_{-\mathbf{k}'\sigma}c_{\mathbf{k}'-\sigma} - \Delta_{\mathbf{k}'\sigma}\Delta^{\star}_{\mathbf{k}'+\mathbf{l}+\mathbf{q}\sigma}. \tag{2}$$

The symbol $\Delta_{\mathbf{k}\sigma}$ is given by: $\Delta_{\mathbf{k}\sigma} \equiv \langle c_{-\mathbf{k}\sigma}c_{\mathbf{k}-\sigma}\rangle$. In the next step, we have substituted (2) into (1). The Hamiltonian can be rewritten as:

$$\begin{aligned} H_{int} \simeq\; & \frac{U}{24N^3}\sum_{\mathbf{k}\mathbf{k}'\mathbf{q}\mathbf{l}\sigma}^{\omega_0}\Delta^{\star}_{\mathbf{k}'+\mathbf{l}+\mathbf{q}\sigma}c_{\mathbf{k}'-\sigma}h^{(2)}_{\mathbf{k}\mathbf{l}\sigma}c_{-\mathbf{k}'\sigma} - \frac{U}{24N^3}\sum_{\mathbf{k}\mathbf{k}'\mathbf{q}\mathbf{l}\sigma}^{\omega_0}\Delta_{\mathbf{k}'\sigma}c^{\dagger}_{\mathbf{k}'+\mathbf{l}+\mathbf{q}-\sigma}h^{(2)}_{\mathbf{k}\mathbf{l}\sigma}c^{\dagger}_{-\mathbf{k}'-\mathbf{l}-\mathbf{q}\sigma} \\ +\; & \frac{U}{24N^3}\sum_{\mathbf{k}\mathbf{k}'\mathbf{q}\mathbf{l}\sigma}^{\omega_0}\Delta^{\star}_{\mathbf{k}'+\mathbf{l}+\mathbf{q}\sigma}\Delta_{\mathbf{k}'\sigma}h^{(2)}_{\mathbf{k}\mathbf{l}\sigma}, \end{aligned} \tag{3}$$

where: $h^{(2)}_{\mathbf{k}\mathbf{l}\sigma} \equiv c^{\dagger}_{-\mathbf{k}+\mathbf{l}-\sigma}c^{\dagger}_{\mathbf{k}-\mathbf{l}\sigma}c_{\mathbf{k}\sigma}c_{-\mathbf{k}-\sigma}$. We notice that the linear terms with reference to the anomalous thermal average ($\Delta_{\mathbf{k}\sigma}$) can exist only on this stage of the presented approximation.

The operator (3) has still very complicated form (the first and second terms comprise the six fermion operators). In order to solve the problem we have simplified the Hamiltonian (3) again:

$$h^{(2)}_{\mathbf{k}\mathbf{l}\sigma} \simeq \Delta_{-\mathbf{k}\sigma}c^{\dagger}_{-\mathbf{k}+\mathbf{l}-\sigma}c^{\dagger}_{\mathbf{k}-\mathbf{l}\sigma} + \Delta^{\star}_{-\mathbf{k}+\mathbf{l}\sigma}c_{\mathbf{k}\sigma}c_{-\mathbf{k}-\sigma} - \Delta_{-\mathbf{k}\sigma}\Delta^{\star}_{-\mathbf{k}+\mathbf{l}\sigma}. \tag{4}$$

Joining the expressions (3) and (4), we have obtained the sum of the terms, which are proportional to $\Delta^2_{\mathbf{k}\sigma}$, $\Delta^3_{\mathbf{k}\sigma}$ or $\Delta^4_{\mathbf{k}\sigma}$. Next, long in the form but straightforward calculations (in the presented mean-field scheme) give:

$$\begin{aligned} H_{int} \equiv\; & \frac{U}{24N^3}\sum_{\mathbf{k}\mathbf{k}'\mathbf{q}\mathbf{l}\sigma}^{\omega_0}\Delta_{\mathbf{k}\sigma}\Delta_{\mathbf{k}'\sigma}\Delta^{\star}_{\mathbf{k}'+\mathbf{l}+\mathbf{q}\sigma}\Delta^{\star}_{\mathbf{k}+\mathbf{l}\sigma} \\ +\; & \frac{U}{24N^3}\sum_{\mathbf{k}\mathbf{q}\mathbf{l}\sigma}^{\omega_0}\Delta_{\mathbf{k}\sigma}\Delta^{\star}_{\mathbf{k}+\mathbf{l}+\mathbf{q}\sigma}\left(c^{\dagger}_{-\mathbf{k}-\mathbf{l}\sigma}c_{-\mathbf{k}-\mathbf{l}\sigma} + c_{-\mathbf{k}-\mathbf{q}\sigma}c^{\dagger}_{-\mathbf{k}-\mathbf{q}\sigma}\right) \\ -\; & \frac{U}{12N^3}\sum_{\mathbf{k}\mathbf{k}'\mathbf{q}\mathbf{l}\sigma}^{\omega_0}\Delta_{\mathbf{k}\sigma}\Delta^{\star}_{\mathbf{k}'+\mathbf{l}\sigma}c_{-\mathbf{k}'\sigma}c_{\mathbf{k}'-\sigma}c^{\dagger}_{\mathbf{k}+\mathbf{l}+\mathbf{q}-\sigma}c^{\dagger}_{-\mathbf{k}-\mathbf{l}-\mathbf{q}\sigma}. \end{aligned} \tag{5}$$

On the basis of the expression (5), we see that in the obtained Hamlitonian exist only the terms, which are proportional to $\Delta^2_{\mathbf{k}\sigma}$ or $\Delta^4_{\mathbf{k}\sigma}$ (all operators with $\Delta^3_{\mathbf{k}\sigma}$ have reduced each other mutually). We notice that the first term in Eq. (5) can be neglected because it does not include the operators. In the case when we separate the momentums in the remained expression, the second term also can be neglected. The third term gives the interaction part of the Hamiltonian (13) in the main body of the paper after using the mean-field approximation.

It is clear that the used scheme of the simplification of the Hamiltoniam (1) is relatively simple. However, the presented approach lets us to analyse the physics of the considered system on the quantitative level. Thus, the agreement between obtained theoretical results and the experimental data will be an essential argument that the presented method is correct.
