# Supplementary material for: Pairing Mechanism for the High-TC Superconductivity: Symmetries and Thermodynamic Properties
Source: PLoS One. 2012 Apr 18;7(4):e31873. doi: 10.1371/journal.pone.0031873 (PMC3329537; doi:10.1371/journal.pone.0031873)
Supplement: Appendix S3 — The accuracy of the fold mean-field approximation in the framework of the random phase approximation (RPA) method. (PDF) [file pone.0031873.s003.pdf]

Appendix S3
Supporting information for

Pairing mechanism for the high-$\mathrm{T_C}$ superconductivity: symmetries and thermodynamic properties

Radosław Szczęśniak∗
Institute of Physics, Częstochowa University of Technology, Al. Armii Krajowej 19, 42-200 Częstochowa, Poland
∗ E-mail: szczesni@wip.pcz.pl

On the form of the anomalous Green function

In order to prove that all omitted terms in the anomalous Green function (also the terms linear with reference to Δ in the pairing potential) can be neglected we should consider the general form of the electronic Hamiltonian:

$$H_e \equiv H_0 + H_{BCS} + H_{int}, \tag{1}$$

where:

$$H_0 \equiv \sum_{\mathbf{k}\sigma} \bar{\varepsilon}_{\mathbf{k}} c^{\dagger}_{\mathbf{k}\sigma} c_{\mathbf{k}\sigma}, \tag{2}$$

$$H_{BCS} \equiv -\frac{V}{2N} \sum_{\mathbf{k}_1\mathbf{k}_2\sigma}^{\omega_0} c^{\dagger}_{\mathbf{k}_1-\sigma} c^{\dagger}_{-\mathbf{k}_1\sigma} c_{-\mathbf{k}_2\sigma} c_{\mathbf{k}_2-\sigma}, \tag{3}$$

$$H_{int} \equiv -\frac{U}{24N^3} \sum_{\mathbf{k}_1\sim\mathbf{k}_4\sigma}^{\omega_0} c^{\dagger}_{-\mathbf{k}_1\sigma} c_{-\mathbf{k}_2\sigma} h_{\mathbf{k}_3\mathbf{k}_4\sigma} c^{\dagger}_{\mathbf{k}_1-\sigma} c_{\mathbf{k}_2-\sigma}, \tag{4}$$

and: $h_{\mathbf{k}_3\mathbf{k}_4\sigma} \equiv c^{\dagger}_{\mathbf{k}_3-\sigma} c^{\dagger}_{-\mathbf{k}_3\sigma} c_{-\mathbf{k}_4\sigma} c_{\mathbf{k}_4-\sigma}$. The anomalous Green function is given by:

$$(\omega - \bar{\varepsilon}_{\mathbf{k}}) << c_{\mathbf{k}\uparrow}|c_{-\mathbf{k}\downarrow} >>=<< [c_{\mathbf{k}\uparrow}, H_{BCS}]_- \, |c_{-\mathbf{k}\downarrow} >> + << [c_{\mathbf{k}\uparrow}, H_{int}]_- \, |c_{-\mathbf{k}\downarrow} >> . \tag{5}$$

The first commutator in Eq. (5) is equal to:

$$[c_{\mathbf{k}\uparrow}, H_{BCS}]_- = -\frac{V}{N} \sum_{\mathbf{k}_1}^{\omega_0} c^{\dagger}_{-\mathbf{k}\downarrow} c_{-\mathbf{k}_1\downarrow} c_{\mathbf{k}_1\uparrow}. \tag{6}$$

By using the RPA method one can show that the higher order Green function is connected with the anomalous and normal Green functions [1]:

$$\begin{aligned} -\frac{V}{N} \sum_{\mathbf{k}_1}^{\omega_0} << c^{\dagger}_{-\mathbf{k}\downarrow} c_{-\mathbf{k}_1\downarrow} c_{\mathbf{k}_1\uparrow}|c_{-\mathbf{k}\downarrow} >> \;&\simeq\; -V\frac{1}{N} \sum_{\mathbf{k}_1}^{\omega_0} \left\langle c^{\dagger}_{-\mathbf{k}\downarrow} c_{-\mathbf{k}_1\downarrow} \right\rangle \delta_{\mathbf{k}\mathbf{k}_1} << c_{\mathbf{k}_1\uparrow}|c_{-\mathbf{k}\downarrow} >> \\ &\quad -V\frac{1}{N} \sum_{\mathbf{k}_1}^{\omega_0} \left\langle c_{-\mathbf{k}_1\downarrow} c_{\mathbf{k}_1\uparrow} \right\rangle << c^{\dagger}_{-\mathbf{k}\downarrow}|c_{-\mathbf{k}\downarrow} >> \\ &= -V\frac{\langle n_{-\mathbf{k}\downarrow}\rangle}{N} << c_{\mathbf{k}\uparrow}|c_{-\mathbf{k}\downarrow} >> -V\Delta << c^{\dagger}_{-\mathbf{k}\downarrow}|c_{-\mathbf{k}\downarrow} >>, \end{aligned} \tag{7}$$

where: $\langle n_{\mathbf{k}\sigma}\rangle \equiv \left\langle c^{\dagger}_{\mathbf{k}\sigma} c_{\mathbf{k}\sigma} \right\rangle$. On the basis of Eqs. (5) and (7) we state that the term $-V\frac{\langle n_{-\mathbf{k}\downarrow}\rangle}{N}$ renormalize the electron band energy. However, it can be neglected since: $\frac{\langle n_{-\mathbf{k}\downarrow}\rangle}{N} \to 0$. The second term $(-V\Delta)$ represents the classical BCS result; see also the Green function in Eq. (16) in the main body of the paper.

The presented method of analysis enables the determination of the form of the pairing potential and the self-energy terms which are connected with the additional channel given by Eq. (4). In the first step, we calculate the commutator inside the Green function: $<< \left[c_{\mathbf{k}\uparrow}, H_{int}\right]_{-} | c_{-\mathbf{k}\downarrow} >>$. The result has the form:

$$\begin{aligned}
\left[c_{\mathbf{k}\uparrow}, H_{int}\right]_{-} = & - \frac{U}{24N^3} \sum_{\mathbf{k}_2\mathbf{k}_3\mathbf{k}_4}^{\omega_0} c_{\mathbf{k}_2\uparrow} \left(c^{\dagger}_{\mathbf{k}_3\downarrow} c^{\dagger}_{-\mathbf{k}_3\uparrow} c_{-\mathbf{k}_4\uparrow} c_{\mathbf{k}_4\downarrow} \right) c^{\dagger}_{-\mathbf{k}\downarrow} c_{-\mathbf{k}_2\downarrow} \\
& - \frac{U}{24N^3} \sum_{\mathbf{k}_2\mathbf{k}_3\mathbf{k}_4}^{\omega_0} c^{\dagger}_{-\mathbf{k}\downarrow} c_{-\mathbf{k}_2\downarrow} \left(c^{\dagger}_{\mathbf{k}_3\downarrow} c^{\dagger}_{-\mathbf{k}_3\uparrow} c_{-\mathbf{k}_4\uparrow} c_{\mathbf{k}_4\downarrow} \right) c_{\mathbf{k}_2\uparrow} \\
& - \frac{U}{24N^3} \sum_{\mathbf{k}_1\mathbf{k}_2\mathbf{k}_4}^{\omega_0} c^{\dagger}_{\mathbf{k}_1\downarrow} c_{\mathbf{k}_2\downarrow} \left(c^{\dagger}_{-\mathbf{k}\downarrow} c_{-\mathbf{k}_4\downarrow} c_{\mathbf{k}_4\uparrow} \right) c^{\dagger}_{-\mathbf{k}_1\uparrow} c_{-\mathbf{k}_2\uparrow} \\
& - \frac{U}{24N^3} \sum_{\mathbf{k}_1\mathbf{k}_2\mathbf{k}_4}^{\omega_0} c^{\dagger}_{-\mathbf{k}_1\uparrow} c_{-\mathbf{k}_2\uparrow} \left(c^{\dagger}_{-\mathbf{k}\downarrow} c_{-\mathbf{k}_4\downarrow} c_{\mathbf{k}_4\uparrow} \right) c^{\dagger}_{\mathbf{k}_1\downarrow} c_{\mathbf{k}_2\downarrow},
\end{aligned} \quad (8)$$

where the brackets () in Eq. (8) are introduced in order to lighten the notation.

Next, the Green function $<< \left[c_{\mathbf{k}\uparrow}, H_{int}\right]_{-} | c_{-\mathbf{k}\downarrow} >>$ in analogy to $<< \left[c_{\mathbf{k}\uparrow}, H_{BCS}\right]_{-} | c_{-\mathbf{k}\downarrow} >>$ has been transformed. As a result one can obtain the terms connected with the anomalous and normal Green function (on account of their large number we have not written out the list of them explicitly). On the basis of the calculations presented in Eq.(7), it is easy to understand that only terms proportional to $|\Delta|^2\Delta$ are important, since they do not have the ratio $\frac{\langle n_{\mathbf{k}\sigma}\rangle}{N}$. Finally, we notice that all terms linear with reference to Δ in the pairing potential are unimportant since they comprise $\frac{\langle n_{\mathbf{k}\sigma}\rangle}{N}$; the constant terms with reference to Δ in the pairing potential are proportional to $\left[\frac{\langle n_{\mathbf{k}\sigma}\rangle}{N}\right]^2$.

References

1. Gasser W, Heiner E, Elk K (1999) Greensche Funktionen in Festkörper- und Vielteilchenphysik. Weinheim: VILEY-VCH Verlag GmbH.
