# Supplementary material for: Pairing Mechanism for the High-TC Superconductivity: Symmetries and Thermodynamic Properties
Source: PLoS One. 2012 Apr 18;7(4):e31873. doi: 10.1371/journal.pone.0031873 (PMC3329537; doi:10.1371/journal.pone.0031873)
Supplement: Appendix S4 — The van Hove and generalized mean-field thermodynamic potential. (PDF) [file pone.0031873.s004.pdf]

Appendix S4
Supporting information for

Pairing mechanism for the high-$\mathrm{T_C}$ superconductivity: symmetries and thermodynamic properties

Radosław Szczęśniak*
Institute of Physics, Częstochowa University of Technology, Al. Armii Krajowej 19, 42-200 Częstochowa, Poland
* E-mail: szczesni@wip.pcz.pl

The van Hove and generalized mean-field thermodynamic potential

In the first step, we will calculate the thermodynamic potential in the framework of the BCS van Hove scenario. Next, we will generalize the results for the case $U \neq 0$.

For $U = 0$ the thermodynamic potential is given by:

$$\Omega\left(V^{'}\right) = -\frac{1}{\beta}\ln\left[Z\left(V^{'}\right)\right], \tag{1}$$

where the grand partition function has the form:

$$Z\left(V^{'}\right) \equiv \mathrm{Tr}\left[e^{-\beta\left(H_A - \frac{V^{'}}{2}H_B\right)}\right]. \tag{2}$$

The symbols H_A and H_B denote the following operators:

$$H_A \equiv \sum_{\mathbf{k}\sigma} \overline{\varepsilon}_{\mathbf{k}} c^{\dagger}_{\mathbf{k}\sigma} c_{\mathbf{k}\sigma}, \tag{3}$$

and

$$H_B \equiv \frac{1}{N}\sum^{\omega_0}_{\mathbf{k}\mathbf{k}^{'}\sigma} c^{\dagger}_{\mathbf{k}-\sigma} c^{\dagger}_{-\mathbf{k}\sigma} c_{-\mathbf{k}^{'}\sigma} c_{\mathbf{k}^{'}-\sigma}. \tag{4}$$

The thermodynamic potential is readily found from the expression:

$$\begin{aligned}\frac{\partial\Omega\left(V^{'}\right)}{\partial V^{'}} &= -\frac{1}{\beta Z\left(V^{'}\right)} \\ &\frac{\partial}{\partial V^{'}}\mathrm{Tr}\left[\sum_{j=0}^{+\infty}\frac{1}{j!}\left(-\beta\left(H_A - \frac{V^{'}}{2}H_B\right)\right)^j\right] \\ &= -\frac{1}{2}\left<H_B\right>.\end{aligned} \tag{5}$$

Integrate Eq. (5) from $V^{'} = 0$ to $V^{'} = V$ we obtain:

$$\begin{aligned}\Delta\Omega_V &\equiv \frac{1}{N}\left[\Omega\left(V\right) - \Omega\left(0\right)\right] \\ &= -\frac{1}{2N}\int_0^V dV^{'}\left<H_B\right> \\ &\simeq -\int_0^V dV^{'}\left(\frac{1}{V^{'}}\right)^2 |\Delta_{V^{'}}|^2,\end{aligned} \tag{6}$$

where $\Delta_{V'} \equiv V^{'}\Delta$. The formula (6) may be rewritten as follows:

$$\Delta\Omega_V = \int_0^{\Delta_V} d\Delta_{V'} \left(\Delta_{V'}\right)^2 \frac{d\left(\frac{1}{V'}\right)}{d\Delta_{V'}}. \tag{7}$$

After substituting Eq. (18) in the main body of the paper (for $U = 0$) into expression (7) we find:

$$\begin{aligned} \Delta\Omega_V &= \frac{\Delta_V^2}{V} - 2\int_0^{\omega_0} d\varepsilon \rho\left(\varepsilon\right)\left(E-\varepsilon\right) \\ &+ \frac{4}{\beta}\int_0^{\omega_0} d\varepsilon \rho\left(\varepsilon\right) \ln\left(1+e^{-\beta\varepsilon}\right) \\ &- \frac{4}{\beta}\int_0^{\omega_0} d\varepsilon \rho\left(\varepsilon\right) \ln\left(1+e^{-\beta E}\right). \end{aligned} \tag{8}$$

The first integral in Eq. (8) is given by:

$$\begin{aligned} I_1\left(\Delta_V\right) &\equiv -2\int_0^{\omega_0} d\varepsilon \rho\left(\varepsilon\right)\left(E-\varepsilon\right) \\ &= b_1 \Delta_V^2 \sum_{j=1}^{3} f_j\left(\Delta_V\right), \end{aligned} \tag{9}$$

where:

$$f_1\left(\Delta_V\right) \equiv \left(\frac{\omega_0}{\Delta_V}\right) F_{3,2}\left[\frac{1}{2},\frac{1}{2},\frac{1}{2};\frac{3}{2},\frac{3}{2};-\left(\frac{\omega_0}{\Delta_V}\right)^2\right], \tag{10}$$

$$f_2\left(\Delta_V\right) \equiv \left[\frac{1}{2} - \ln\left(\omega_0\right)\right]\left[\left(\frac{\omega_0}{\Delta_V}\right)^2\left[\sqrt{1+\left(\frac{\Delta_V}{\omega_0}\right)^2} - 1\right] + \operatorname{arcsinh}\left(\frac{\omega_0}{\Delta_{\rm V}}\right)\right], \tag{11}$$

and

$$f_3\left(\Delta_V\right) \equiv \ln\left(b_2\right)\left[\left(\frac{\omega_0}{\Delta_V}\right)^2\left[\sqrt{1+\left(\frac{\Delta_V}{\omega_0}\right)^2} - 1\right] + \ln\left[\left(\frac{\omega_0}{\Delta_V}\right)\left[\sqrt{1+\left(\frac{\Delta_V}{\omega_0}\right)^2} + 1\right]\right]\right]. \tag{12}$$

The symbol $F_{p,q}\left(a;b;z\right)$ denotes the generalized hypergeometric function.

The second integral in Eq. (8) describes the first temperature-dependent correction to the thermodynamic potential in the normal state:

$$I_2\left(T\right) \equiv \frac{4}{\beta}\int_0^{\omega_0} d\varepsilon \rho\left(\varepsilon\right) \ln\left(1+e^{-\beta\varepsilon}\right). \tag{13}$$

The expression (13) can be rewritten by using the partial integration method. Since $\omega_0 >> k_B T$, the obtained integral may be extended to infinity. In this way, we can find:

$$\begin{aligned} I_2\left(T\right) &= -b_1\left[\kappa + \ln\left(b_2\beta\right)\right]\frac{\pi^2}{3\beta^2} \\ &+ b_1\omega_0\left[\ln\left(\omega_0\right) - 1\right]\ln\left(1+e^{-\beta\omega_0}\right)\frac{4}{\beta}. \end{aligned} \tag{14}$$

The number $\kappa \simeq 0.45403$ is defined by:

$$\begin{aligned} \kappa &\equiv 1+\ln(2)+\gamma \\ &+ \frac{3}{\pi^2}\left[\left(\frac{\partial \gamma_1(z)}{\partial z}\right)_{z=1} - \left(\frac{\partial \gamma_1(z)}{\partial z}\right)_{z=\frac{1}{2}}\right], \end{aligned} \tag{15}$$

where the symbol $\gamma_n(z)$ is the generalized Stieltjes constant.

We also notice that, by using the Eq. (7) and Eq. (26) in the main body of the paper it is possible to derive explicit expression for $\Delta\Omega_V$ close to the transition temperature:

$$\Delta\Omega_V = -\frac{b_1}{2p_2(T)}\left[p_1(T) - \frac{1}{b_1 V}\right]^2. \tag{16}$$

The dependence of $\Delta\Omega_V$ on the temperature is shown in Fig. S4.

The calculation of the thermodynamic potential for $U \neq 0$ is more difficult problem. In this case:

$$\Omega\left(V', U'\right) = -\frac{1}{\beta}\ln\left[Z\left(V', U'\right)\right], \tag{17}$$

where the grand partition function has the form:

$$Z\left(V', U'\right) \equiv \mathrm{Tr}\left[e^{-\beta\left(H_A - \frac{V'}{2}H_B - \frac{U'}{24}H_C\right)}\right]. \tag{18}$$

In Eq. (18) the Hamiltonian H_C is given by:

$$H_C \equiv \frac{1}{N^3}\sum_{\mathbf{k}\mathbf{k}'\mathbf{q}\mathbf{l}\sigma} c^{\dagger}_{\mathbf{k}-\mathbf{l}\sigma} c_{\mathbf{k}\sigma} c^{\dagger}_{\mathbf{k}'+\mathbf{l}+\mathbf{q}-\sigma} c_{\mathbf{k}'-\sigma} c^{\dagger}_{-\mathbf{k}'-\mathbf{l}-\mathbf{q}\sigma} c_{-\mathbf{k}'\sigma} c^{\dagger}_{-\mathbf{k}+\mathbf{l}-\sigma} c_{-\mathbf{k}-\sigma}. \tag{19}$$

Now, we consider the total differential of the thermodynamic potential:

$$d\Omega\left(V', U'\right) = \frac{\partial\Omega\left(V', U'\right)}{\partial V'}dV' + \frac{\partial\Omega\left(V', U'\right)}{\partial U'}dU'. \tag{20}$$

By using the method presented for the BCS van Hove scenario we can obtain:

$$\frac{\partial\Omega\left(V', U'\right)}{\partial V'} = -\frac{1}{2}\langle H_B\rangle \simeq -N|\Delta\left(V', U'\right)|^2, \tag{21}$$

and

$$\frac{\partial\Omega\left(V', U'\right)}{\partial U'} = -\frac{1}{24}\langle H_C\rangle \simeq -\frac{N}{12}|\Delta\left(V', U'\right)|^4. \tag{22}$$

Finally, the general evaluation of the thermodynamic potential requires the numerical analysis of the expression:

$$\begin{aligned} \Delta\Omega_{VU} &\equiv \frac{1}{N}\left[\Omega(V,U) - \Omega(0,0)\right] \\ &\simeq -\int_{(0,0)}^{(V,U)} |\Delta\left(V', U'\right)|^2 dV' + \frac{1}{12}|\Delta\left(V', U'\right)|^4 dU' \\ &= -\int_0^1 dx\left[V|\Delta(Vx, Ux)|^2 + \frac{U}{12}|\Delta(Vx, Ux)|^4\right]. \end{aligned} \tag{23}$$
