# Supplementary material for: Pairing Mechanism for the High-TC Superconductivity: Symmetries and Thermodynamic Properties
Source: PLoS One. 2012 Apr 18;7(4):e31873. doi: 10.1371/journal.pone.0031873 (PMC3329537; doi:10.1371/journal.pone.0031873)
Supplement: Appendix S5 — The lists of the experimental values of the thermodynamic parameters for the selected high- superconductors. (PDF) [file pone.0031873.s005.pdf]

Appendix S5
Supporting information for

Pairing mechanism for the high-$\mathrm{T_C}$ superconductivity: symmetries and thermodynamic properties

Radosław Szczęśniak∗
Institute of Physics, Częstochowa University of Technology, Al. Armii Krajowej 19, 42-200 Częstochowa, Poland
∗ E-mail: szczesni@wip.pcz.pl

Experimental values of T_C and the low-temperature superconducting gap

In the Appendix we provide the list of the thermodynamic parameters values of high-T_C superconductors which have been obtained experimentally (Tab. 1 S5, Tab. 2 S5, Tab. 3 S5, Tab. 4 S5, Tab. 5 S5). In particular, we have collected the data for T_C and the energy gap $\Delta^{(0)}_{tot}$. We have also determined the doping level or stoichiometry of the materials and the values of R_1 parameter.
