# Supplementary material for: Pairing Mechanism for the High-TC Superconductivity: Symmetries and Thermodynamic Properties
Source: PLoS One. 2012 Apr 18;7(4):e31873. doi: 10.1371/journal.pone.0031873 (PMC3329537; doi:10.1371/journal.pone.0031873)
Supplement: Table S1 — The experimental data for YBa2Cu3O7−y (YBCO). (PDF) [file pone.0031873.s007.pdf]

Table 1 S5
Supporting information for

Pairing mechanism for the high-T_C superconductivity: symmetries and thermodynamic properties

Radosław Szczęśniak∗
Institute of Physics, Częstochowa University of Technology, Al. Armii Krajowej 19, 42-200 Częstochowa, Poland
∗ E-mail: szczesni@wip.pcz.pl

Table 1. The experimental data for $YBa_2Cu_3O_{7-y}$ (YBCO).

Type	T_C (K)	$\Delta_{tot}^{(0)}$ (meV)	R_1	Ref.
$p = 0.079$	44	66	34.81	[1]
$p = 0.111$	62	71	26.58	
$p = 0.166$	93.5	50	12.41	
$p = 0.184$	89	37	9.65	
$p = 0.106$	60	58 ± 8.8	22.37 ± 3.39	[2]
$p = 0.137$	80	45 ± 4.9	13.04 ± 1.40	
$p = 0.175$	92	34 ± 3.1	8.55 ± 0.77	
$p = 0.099$	57.4	56.8	22.96	[3]
$p = 0.110$	61.8	54.2	20.36	
$p = 0.140$	83	37.6	10.52	
$p = 0.160$	93.2	33.8	8.42	
$p = 0.080$	46.2	66.3	33.27	[4]
$p = 0.096$	56.3	71	29.3	
$p = 0.160$	93.2	49.9	12.42	
$p = 0.184$	88.9	37.2	9.7	
$p = 0.086$	51.6	12.5	5.62	[5], [6]
$p = 0.096$	56.3	14	5.77	
$p = 0.101$	58	16.5	6.6	
$p = 0.171$	93	20.5	5.12	
$p = 0.106$	60	~ 20	7.7	[7]
$p = 0.159$	92.9	18	4.5	
$p = 0.149$	89	20	5.2	[8]
$p = 0.153$	91	24-32	7.1	[9]
$p = 0.153$	91	$\sim 25^a$	6.4	
$p = 0.156$	92	30 ± 8	7.6	[10]
$p = 0.156$	92	20	5	[11]

[a]Tunneling on electrical field etched surface.

References

1. Sutherland M, Hawthorn DG, Hill RW, Ronning F, Wakimoto S, et al. (2003) Thermal conductivity across the phase diagram of cuprates: Low-energy quasiparticles and doping dependence of the superconducting gap. Phys Rev B 67: 174520-1-174520-11.

2. Nakayama K, Sato T, Terashima K, Arakane T, Takahashi T, et al. (2009) Doping dependence of the gap anisotropy of the high-temperature $yba_2cu_3o_{7-\delta}$ superconductor. Phys Rev B 79: 140503(R)-1-140503(R)-4.

3. Kaminski A, Rosenkranz S, Fretwell HM, Mesot J, Randeria M, et al. (2004) Identifying the background signal in angle-resolved photoemission spectra of high-temperature cuprate superconductors. Phys Rev B 69: 212509-1-212509-4.

4. Plate M, Mottershead JDF, Elfimov IS, Peets DC, Liang R, et al. (2005) Fermi surface and quasiparticle excitations of overdoped $tl_2ba_2cuo_{6+\delta}$. Phys Rev Lett 95: 077001-1-077001-4.

5. Morr DK, Pines D (1998) The resonance peak in cuprate superconductors. Phys Rev Lett 81: 1086-1089.

6. Fong HF, Keimer B, Milius DL, Aksay IA (1997) Superconductivity-induced anomalies in the spin excitation spectra of underdoped $yba_2cu_3o_{6+x}$. Phys Rev Lett 78: 713-716.

7. Yeh NC, Chen CT, Hammer G, Mannhart J, Schmehl A, et al. (2001) Evidence of doping-dependent pairing symmetry in cuprate superconductors. Phys Rev Lett 87: 087003-1-087003-4.

8. Born V, Jooss C, Freyhardt HC (2002) Scanning tunneling spectroscopy of optimally doped and underdoped $yba_2cu_3o_x$ thin films. Physica C 382: 224-232.

9. Murakami H, Asaoka H, Sakai K, Ito T, Tonouchi M (2001) Lt-stm/sts observations on electrical field etched surfaces of $yba_2cu_3o_{7-\delta}$ single crystal. Appl Surf Sci 175-176: 306-311.

10. Edwards HL, Markert JT, de Lozanne AL (1992) Energy gap and surface structure of $yba_2cu_3o_{7-x}$ probed by scanning tunneling microscopy. Phys Rev Lett 69: 2967-2970.

11. Edwards HL, Derro DJ, Barr AL, Markert JT, de Lozanne AL (1995) Spatially varying energy gap in the cuo chains of $yba_2cu_3o_{7-x}$ detected by scanning tunneling spectroscopy. Phys Rev Lett 75: 1387-1390.
