# Supplementary material for: Pairing Mechanism for the High-TC Superconductivity: Symmetries and Thermodynamic Properties
Source: PLoS One. 2012 Apr 18;7(4):e31873. doi: 10.1371/journal.pone.0031873 (PMC3329537; doi:10.1371/journal.pone.0031873)
Supplement: Table S2 — The experimental data for Bi2Sr2CaCu2O8+y (Bi2212). (PDF) [file pone.0031873.s008.pdf]

Table 2 S5
Supporting information for

Pairing mechanism for the high-T_C superconductivity: symmetries and thermodynamic properties

Radosław Szczęśniak*
Institute of Physics, Częstochowa University of Technology, Al. Armii Krajowej 19, 42-200 Częstochowa, Poland
* E-mail: szczesni@wip.pcz.pl

Table 1. The experimental data for $Bi_2Sr_2CaCu_2O_{8+y}$ (Bi2212).

Type	T_C (K)	$\Delta^{(0)}_{tot}$ (meV)	R_1	Ref.
$p = 0.125$	83	44 ± 2	12.30 ± 0.56	[1], [2]
$p = 0.160$	92.2	41.5 ± 2	10.45 ± 0.5	
$p = 0.208$	74.3	34 ± 2	10.62 ± 0.62	
$p = 0.229$	56	21 ± 2	8.7 ± 0.83	
$p = 0.131$	86	32.2 ± 1.1	8.7 ± 0.3	[3]
$p = 0.198$	81	28.6 ± 1.4	8.2 ± 0.4	
$p = 0.189$	86 ± 4	25 ± 1	6.7 ± 0.5	[4]
$p = 0.186$	87 ± 1	26 ± 1	8.2 ± 0.3	[5]
$p = 0.165$	92 ± 3	35 ± 1	8.9 ± 0.7	
$p = 0.134$	~ 87	~ 45	~ 12	
$p = 0.127$	84 ± 4	32 ± 0.5	8.8 ± 0.6	
$p = 0.123$	82 ± 4	33.5 ± 0.5	9.4 ± 0.6	
$p = 0.160$	92.5	32.5	8.15	[6]
$p = 0.181$	89	25.8	6.73	
$p = 0.106$	70	38	12.6	[7]
$p = 0.191$	85	30 ± 2	8.2 ± 0.5	
$p = 0.103$	67	39.8	13.79	[8]
$p = 0.122$	80	35.9	10.42	
$p = 0.086$	50.9	64.5	29.39	[9], [10].
$p = 0.089$	54.2	61.2	26.21	
$p = 0.110$	73.2	47.8	15.16	
$p = 0.115$	76.8	50.1	15.14	
$p = 0.121$	80.6	46.1	13.27	
$p = 0.133$	86.7	43.5	11.65	
$p = 0.161$	92.2	37.5	9.43	
$p = 0.186$	87	31	8.28	
$p = 0.193$	83.8	36.6	10.14	
$p = 0.201$	79.3	25.8	7.56	
$p = 0.205$	76.6	34	10.32	
$p = 0.215$	69	27.2	9.13	
$p = 0.100$	63	40	14.7	[11]
$p = 0.130$	85	33	9.0	
$p = 0.190$	85	26	7.1	
$p = 0.100$	60	36 ± 2	13.9	[12]
$p = 0.140$	82	34 ± 2	9.6	
$p = 0.160$	88	32 ± 2	8.4	
$p = 0.210$	81	27 ± 2	7.7	
$p = 0.110$	65	62	22.1	[13]
$p = 0.130$	75	48 ± 1	14.9	
$p = 0.150$	79	43 ± 1	12.6	
$p = 0.180$	89	36 ± 1	9.4	
$p = 0.190$	89	33 ± 1	8.6	
$p = 0.110$	67	55 ± 15	19.1	[14]
$p = 0.130$	85	45 ± 12	12.3	
$p = 0.160$	89	40 ± 10	10.4	
$p = 0.180$	89	35 ± 7	9.1	
$p = 0.220$	64	22 ± 5	8	
$p = 0.120$	78	50.2	14.9	[15]
$p = 0.160$	92	43.7	11	
$p = 0.190$	85	36.7	10	
$p = 0.120$	80	42 ± 2	12.2	[16]
$p = 0.120$	81	40	11.5	[17]

References

1. Renner C, Revaz B, Genoud JY, Kadowaki K, Fischer O (1998) Pseudogap precursor of the superconducting gap in under- and overdoped $bi_2sr_2cacu_2o_{8+\delta}$. Phys Rev Lett 80: 149-152.

2. Renner C, Revaz B, Kadowaki K, Maggio-Aprile I, Fischer O (1998) Observation of the low temperature pseudogap in the vortex cores of $bi_2sr_2cacu_2o_{8+\delta}$. Phys Rev Lett 80: 3606-3609.

3. Hoffmann A, Lemmens P, Winkeler L, Guntherodt G (1995) The pairing mechanism in htsc investigated by electronic raman-scattering. J Low Temp Phys 99: 201-203.

4. Ponomarev YG, Timergaleev NZ, Zabezhaylov AO, Uk KK, Lorenz MA, et al. (2000) Conference Series-Institute of Physics 2: 167.

5. Oki T, Tsuda N, Shimada D (2001) Superconducting energy gap of underdoped and overdoped $bi_2sr_2cacu_2o_8$. Physica C 353: 213-220.

6. Krasnov VM, Yurgens A, Winkler D, Delsing P, Claeson T (2000) Evidence for coexistence of the superconducting gap and the pseudogap in bi-2212 from intrinsic tunneling spectroscopy. Phys Rev Lett 84: 5860-5863.

7. Gupta AK, Ng KW (1998) ab-plane tunneling spectroscopy of underdoped $bi_2sr_2cacu_2o_y$. Phys Rev B 58: R8901-R8904.

8. Kanigel A, Chatterjee U, Randeria M, Norman MR, Souma S, et al. (2007) Protected nodes and the collapse of fermi arcs in high-t_C cuprate superconductors. Phys Rev Lett 99: 157001-1-157001-4.

9. Campuzano JC, Ding H, Norman MR, Fretwell HM, Randeria M, et al. (1999) Electronic spectra and their relation to the (π, π) collective mode in high-t_C superconductors. Phys Rev Lett 83: 3709-3712.

10. Tanaka K, Lee WS, Lu DH, Fujimori A, Fujii T, et al. (2006) Distinct fermi-momentum-dependent energy gaps in deeply underdoped bi2212. Science 314: 1910-1913.

11. Nakano T, Momono N, Oda M, Ido M (1998) Correlation between the doping dependences of superconducting gap magnitude $2\delta_0$ and pseudogap temperature t^* in high-t_C cuprates. J Phys Soc Jpn 67: 2622-2625.

12. Oda M, Hoya K, Kubota R, Manabe C, Momono N, et al. (1997) Strong pairing interactions in the underdoped region of $bi_2sr_2cacu_2o_{8+\sigma}$. Physica C 281: 135-142.

13. McElroy K, Lee DH, Hoffmann JE, Lang KM, Lee J, et al. (2005) Coincidence of checkerboard charge order and antinodal state decoherence in strongly underdoped superconducting $bi_2sr_2cacu_2o_{8+\delta}$. Phys Rev Lett 94: 197005-1-197005-4.

14. Matsuda A, Fujii T, Watanabe T (2003) Gap inhomogeneity, phase separation and a pseudogap in $bi_2sr_2cacu_2o_{8+\delta}$. Physica C 388-389: 207-208.

15. Hoffman JE, Hudson EW, Lang KM, Madhavan V, Eisaki H, et al. (2002) A four unit cell periodic pattern of quasi-particle states surrounding vortex cores in $bi_2sr_2cacu_2o_{8+\delta}$. Science 295: 466-469.

16. Howald C, Fournier P, Kapitulnik A (2001) Inherent inhomogeneities in tunneling spectra of $bi_2sr_2cacu_2o_{8-x}$ crystals in the superconducting state. Phys Rev B 64: 100504(R)-1-100504(R)-4.

17. Murakami H, Aoki R (1995) Observation of multi-stage superconducting gap states in $bi_2sr_2cacu_2o_x$ crystal surface by lt-stm/sts. J Phys Soc Jpn 64: 1287-1292.
