# Supplementary material for: Pairing Mechanism for the High-TC Superconductivity: Symmetries and Thermodynamic Properties
Source: PLoS One. 2012 Apr 18;7(4):e31873. doi: 10.1371/journal.pone.0031873 (PMC3329537; doi:10.1371/journal.pone.0031873)
Supplement: Table S3 — The experimental data for Bi2Sr2Ca2Cu3O10+y (Bi2223). (PDF) [file pone.0031873.s009.pdf]

Table 3 S5
Supporting information for

Pairing mechanism for the high-T_C superconductivity: symmetries and thermodynamic properties

Radosław Szczęśniak∗
Institute of Physics, Częstochowa University of Technology, Al. Armii Krajowej 19, 42-200 Częstochowa, Poland
∗ E-mail: szczesni@wip.pcz.pl

Table 1. The experimental data for $Bi_2Sr_2Ca_2Cu_3O_{10+y}$ **(Bi2223).**

Type	T_C (K)	$\Delta_{tot}^{(0)}$ (meV)	R_1	Ref.
OP	110 ± 5	36 ± 1.6	7.6 ± 0.5	[1]
UD	109	60 ± 3	12.8	[2]
OP	111	45 ± 7	9.4	
OP	109	~ 37	~ 7.88	[3]

References

1. Ponomarev YG, Timergaleev NZ, Zabezhaylov AO, Uk KK, Lorenz MA, et al. (2000) Conference Series-Institute of Physics 2: 167.

2. Kugler M, de Castro GL, Giannini E, Piriou A, Manuel AA, et al. (2006) Scanning tunneling spectroscopy on $bi_2sr_2ca_2cu_3o_{10+\delta}$ single crystals. J Phys Chem Solids 67: 353-356.

3. Masui T, Limonov M, Uchiyama H, Lee S, Tajima S, et al. (2003) Raman study of carrier-overdoping effects on the gap in high-t_C superconducting cuprates. Phys Rev B 68: 060506(R)-1-060506(R)-4.
