# Supplementary material for: Pairing Mechanism for the High-TC Superconductivity: Symmetries and Thermodynamic Properties
Source: PLoS One. 2012 Apr 18;7(4):e31873. doi: 10.1371/journal.pone.0031873 (PMC3329537; doi:10.1371/journal.pone.0031873)
Supplement: Table S4 — The experimental data for NdBa2Cu3O7−y (NdBCO). (PDF) [file pone.0031873.s010.pdf]

Table 4 S5
Supporting information for

Pairing mechanism for the high-$\mathrm{T_C}$ superconductivity: symmetries and thermodynamic properties

Radosław Szczęśniak∗
Institute of Physics, Częstochowa University of Technology, Al. Armii Krajowej 19, 42-200 Częstochowa, Poland
∗ E-mail: szczesni@wip.pcz.pl

Table 1. The experimental data for $\mathrm{NdBa_2Cu_3O_{7-y}}$ (NdBCO).

Type	T_C (K)	$\Delta_{tot}^{(0)}$ (meV)	R_1	Ref.
y=0	95	30	7.3	[1]

References

1. Nishiyama N, Kinoda G, Shibata S, Hasegawa T, Koshizuka N, et al. (2002) Low temperature scanning tunneling spectroscopy studies of high j_C $ndba_2cu_3o_{7-\delta}$ single crystals. J Supercond Nov Magn 15: 351-354.
