# Supplementary material for: Pairing Mechanism for the High-TC Superconductivity: Symmetries and Thermodynamic Properties
Source: PLoS One. 2012 Apr 18;7(4):e31873. doi: 10.1371/journal.pone.0031873 (PMC3329537; doi:10.1371/journal.pone.0031873)
Supplement: Table S5 — The experimental data for Pr2−xCexCuO4−y (PCCO). (PDF) [file pone.0031873.s011.pdf]

Table 5 S5
Supporting information for

Pairing mechanism for the high-T_C superconductivity: symmetries and thermodynamic properties

Radosław Szczęśniak*
Institute of Physics, Częstochowa University of Technology, Al. Armii Krajowej 19, 42-200 Częstochowa, Poland
* E-mail: szczesni@wip.pcz.pl

Table 1. The experimental data for $Pr_{2-x}Ce_xCuO_{4-y}$ (PCCO).

Type	T_C (K)	$\Delta^{(0)}_{tot}$ (meV)	R_1	Ref.
x=0.13	12.2	6.3 ± 0.6	11.99 ± 1.2	[1]
x=0.15	21.6	4.6 ± 0.3	4.95 ± 0.36	
x=0.17	11.8	1.8 ± 0.2	3.46 ± 0.45	
x=0.15	21	(4.3-5.4)	(4.7-6)	[2]
x=0.17	15	(3-3.6)	(4.7-5.6)	
x=0.15	19 ± 1	3.25	4 ± 0.4	[3]
x=0.16	16 ± 1	2.6	3.8 ± 0.5	
x=0.17	13 ± 1	1.3	2.3 ± 1.3	
x=0.18	11 ± 1	1.0	2.1 ± 1.5	
x=0.19	8 ± 0.4	0.9	2.6 ± 0.9	
x=0.15	20	4.3	5	[4]
x=0.13	10	6.5	15.09	[5]
x=0.15	23	4.4	4.4	
x=0.17	13	1.75	3.12	

References

1. Biswas A, Fournier P, Qazilbash MM, Smolyaninova VN, Balci H, et al. (2002) Evidence of a *d*- to *s*-wave pairing symmetry transition in the electron-doped cuprate superconductor $pr_{2-x}ce_xcuo_4$. Phys Rev Lett 88: 207004-1-207004-4.

2. Zimmers A, Lobo RM, Bontemps N, Homes CC, Barr MC, et al. (2004) Infrared signature of the superconducting state in $pr_{2-x}ce_xcuo_4$. Phys Rev B 70: 132502-1-132502-4.

3. Dagan Y, Beck R, Greene RL (2007) Dirty superconductivity in the electron-doped cuprate $pr_{2-x}ce_xcuo_{4-\delta}$: Tunneling study. Phys Rev Lett 99: 147004-1-147004-4.

4. Homes CC, Lobo RPSM, Fournier P, Zimmers A, Greene RL (2006) Optical determination of the superconducting energy gap in electron-doped $pr_{1.85}ce_{0.15}cuo_4$. Phys Rev B 74: 214515-1-214515-8.

5. Fournier P, Greene RL (2003) Doping dependence of the upper critical field of electron-doped $pr_{2-x}ce_xcuo_4$ thin films. Phys Rev B 68: 094507-1-094507-9.
